# Human iPSC-derived microglia carrying the LRRK2-G2019S mutation show a Parkinson’s disease related transcriptional profile and function

**DOI:** 10.1038/s41598-023-49294-9

**Published:** 2023-12-13

**Authors:** Sohvi Ohtonen, Luca Giudice, Henna Jäntti, Mohammad Feroze Fazaludeen, Anastasia Shakirzyanova, Mireia Gómez-Budia, Nelli-Noora Välimäki, Jonna Niskanen, Nea Korvenlaita, Ilkka Fagerlund, Jari Koistinaho, Mahmood Amiry-Moghaddam, Ekaterina Savchenko, Laurent Roybon, Šárka Lehtonen, Paula Korhonen, Tarja Malm

**Affiliations:** 1 A.I. Virtanen Institute for Molecular Sciences, University of Eastern Finland, Kuopio, Finland; 2 Neuroscience Center, University of Helsinki, Helsinki, Finland; 3 Division of Anatomy, Department of Molecular Medicine, Institute of Basic Medical Sciences, University of Oslo, Oslo, Norway; 4 Stem Cell Laboratory for CNS Disease Modeling, Department of Experimental Medical Science, Lund University, Lund, Sweden; 5 Present Address: F.M. Kirby Neurobiology Center, Boston Children’s Hospital, Boston, MA USA; 6 Present Address: Stanley Center for Psychiatric Research, Broad Institute of MIT and Harvard, Cambridge, MA USA; 7 Present Address: Department of Neurodegenerative Science, The MiND Program, Van Andel Institute, Grand Rapids, MI USA

**Keywords:** Neuroimmunology, Cellular neuroscience

## Abstract

LRRK2-G2019S is one of the most common Parkinson’s disease (PD)-associated mutations and has been shown to alter microglial functionality. However, the impact of LRRK2-G2019S on transcriptional profile of human induced pluripotent stem cell-derived microglia-like cells (iMGLs) and how it corresponds to microglia in idiopathic PD brain is not known. Here we demonstrate that LRRK2-G2019S carrying iMGL recapitulate aspects of the transcriptional signature of human idiopathic PD midbrain microglia. LRRK2-G2019S induced subtle and donor-dependent alterations in iMGL mitochondrial respiration, phagocytosis and cytokine secretion. Investigation of microglial transcriptional state in the midbrains of PD patients revealed a subset of microglia with a transcriptional overlap between the in vitro PD-iMGL and human midbrain PD microglia. We conclude that LRRK2-G2019S iMGL serve as a model to study PD-related effects in human microglia.

## Introduction

Parkinson’s disease (PD) is among the most common neurodegenerative diseases. Pathological hallmarks of PD include a progressive loss of dopaminergic neurons in the midbrain, accumulation of α-synuclein aggregates and formation of Lewy bodies. In addition to these specific hallmarks, PD involves a particular microglia-mediated inflammatory state of the brain^1^. Microglia, which are thought to arise from extra-embryonic erythromyeloid progenitors from the yolk sac^2^, are dynamic guardians of the brain. These unique cells carry out immune surveillance and participate in the development and maintenance of neuronal health^3,4^. However, in patients with neurodegenerative diseases, microglia acquire disease-related states, which can shift from beneficial to pernicious. Despite extensive research, the mechanisms underlying this shift and their contribution to the disease pathogenesis are poorly understood.

In 2004, the association of specific mutations in Leucine-rich repeat kinase 2 (LRRK2) with PD was discovered^5,6^. LRRK2 is a large, multifunctional protein that possesses two distinct enzymatic domains: a GTPase and a serine/threonine kinase domains^7^. Although all the diverse cellular functions of LRRK2 are yet to be discovered, the kinase domain of LRRK2 has been shown to regulate protein activity by autophosphorylation^8–10^ and by phosphorylation of a subset of Rab GTPases^11,12^. LRRK2 has been connected to several vital cellular processes such as autophagy, vesicle trafficking and mitochondrial functions^13–16^, suggesting that alterations in the proper function of LRRK2 may lead to detrimental consequences.

LRRK2 is expressed in many human peripheral immune cell subsets, including monocytes and macrophages^17–19^ and in different human induced pluripotent stem cell (iPSC)-derived immune cell models, such as iPSC-derived macrophages and microglia^20–22^. Furthermore, a recent single cell sequencing study confirms that LRRK2 is expressed in microglia in the human brain^22^. Expression of LRRK2 is induced by inflammatory stimuli following IFNγ exposure^17–19,21,23^, linking LRRK2 expression with immune response.

The most common PD-associated mutation is LRRK2-G2019S^24^, where the glycine is substituted by a serine, leading to increased kinase activity^25^. Rodent models carrying LRRK2-G2019S show a variety of PD linked alterations, such as loss of dopaminergic neurons, and defects in mitochondrial functions and autophagy, depending on the model^26,27^. To assess the LRRK2-G2019S mutation in specific brain cell types in vitro, different iPSC models have been utilized. LRRK2-G2019S has been reported to impair neurite length and mitochondrial functions in iPSC-derived dopaminergic neurons and cause alterations in mitochondria, metabolism, and gene expression in iPSC-derived astrocytes^28^. In iPSC-microglia, LRRK2-G2019S is described to increase phagocytosis and motility and alter LPS-stimulation induced cytokine secretion^21^.

Here we show that human iPSC-derived microglia-like cells (iMGL) carrying the LRRK2-G2019S mutation exhibit a transcriptional profile that recapitulates features of microglia in the human PD brain. On the functional level, mutation-carrying iMGL show alterations in mitochondrial respiration, phagocytosis, and cytokine secretion in a line-dependent manner. Finally, the investigation of human PD midbrain with spatial transcriptomics shows that a subset of microglia from human PD midbrain follow the PD-microglia signature defined by the transcriptional overlap between the in vitro PD-iMGL and the human midbrain PD-microglia characterized by snSEQ.

## Results

### RNA sequencing reveals LRRK2-G2019S mutation induced changes in iMGL

**Figure 1:**
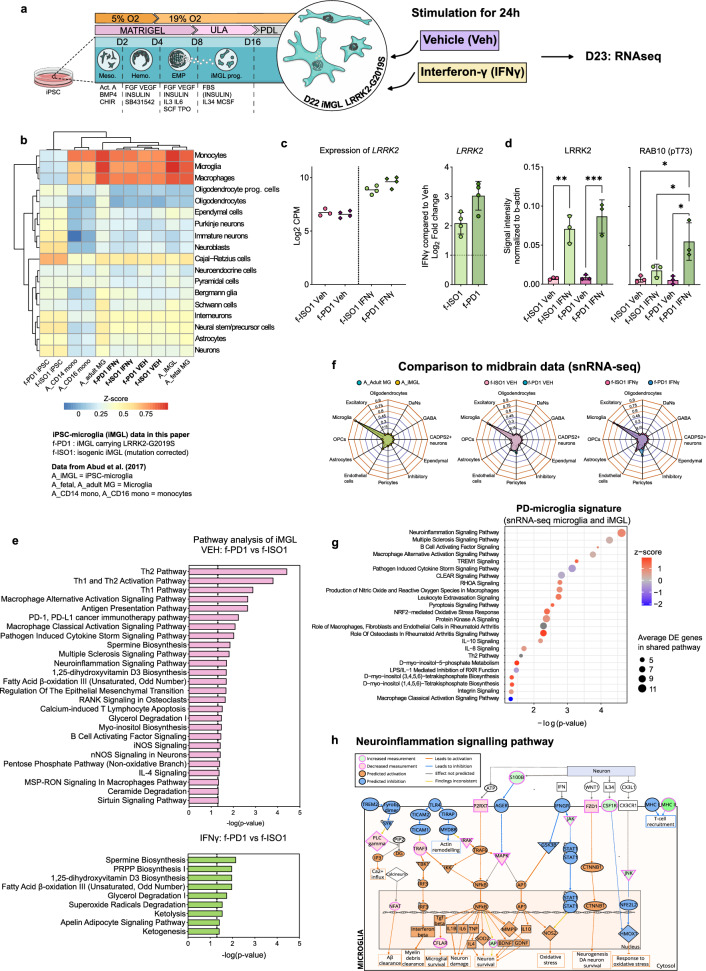
Transcriptional profile of PD-iMGL carrying LRRK2-G2019S and shared PD-microglial expression signature (**a**) Graphical presentation of the differentiation protocol of iPSC-derived microglial-like cells (iMGL). iPSCs are guided to iMGL by the introduction of different cytokines and environmental factors. iPSCs differentiate towards mesoderm (meso.) and hemangioblast (hemo.) in hypoxic conditions. Emerging erythromyeloid progenitors (EMP) are collected and further differentiated in ultra-low attachment (ULA) culture vessels. For experiments, iMGL progenitors are seeded on Poly-d-lysine (PDL) coated culture vessels and the cells are matured for one week before functional analysis. Impact of LRRK2-G2019S in iMGL was studied with RNAseq of vehicle and interferon-γ (IFNγ) stimulated iMGL. (**b**) Cell type signature analysis of our iMGL and (iPSC-)microglia and monocytes from Abud et al. (2017) dataset^29^ showing high correlation of microglial cell type-specific expression profiles. (**c**) iMGL express LRRK2 and the expression is increased by IFNγ. Expression of LRRK2 in iMGL as Log2 CPM (count per million) and Log2 Fold change LRRK2 expression in IFNγ stimulated iMGL. (**d**) Western blot analysis of LRRK2 and phosphorylated RAB10 (pT73) in vehicle and IFNγ stimulated iMGL. n = 3 batches, data are presented as mean ± SD. Two-way Anova with Sidak ´s multiple comparison test, *p < 0.05, **p < 0.01, ***p < 0.001. (**e**) Ingenuity pathway analysis (IPA) how functional annotation of DEGs between f-PD1 and f-ISO1 iMGL. (**f**) Comparison of iMGL transcriptional profile to different cell types extracted from snRNA-SEQ of midbrain^30^, showing that that the majority our iMGL resemble the native microglia at the transcriptional level. (**g**) Shared pathways describing a PD-microglial profile analysed from DEGs between f-PD1/f-ISO1 iMGL & PD and Control microglia. (**h**) Simplified neuroinflammation pathway identified by IPA. For iMGL, n = 3–4 batches of iMGL, with 2 million cells used for each batch (subpanel b–f), n = 2 independently collected batches for both iPSC genotypes (subpanel b). For snSEQ, more details in the original publications^30^, n = 6 controls (22,433 nuclei in total), n = 5 idiopathic PD patients (19 002 nuclei in total), (subpanels e,f).

To identify the molecular pathways that are altered due to the G2019S mutation in our iMGL model, we performed RNAseq analysis on LRRK2-G2019S carrying iMGL and their isogenic controls. As previous studies suggest the role of IFNγ as a regulator for *LRRK2*^17–19,21,23^, we included IFNγ as a inflammatory stimuli for iMGL in our dataset (Fig. 1a). Enrichment analysis heatmap (Fig. 1b) showed a resemblance in the transcriptional profiles between our iMGL samples and the different microglia samples published in the dataset by Abud et al.^29^ in terms of microglia-specific markers and cell type composition. Similarity analysis of the entire gene expression profiles (Supp. Fig. 1b) highlights that our iMGL are more similar to Abud et al.'s microglia than to monocytes. In addition, *LRRK2* is prominently expressed in our iMGL and is further increased by IFNγ exposure (Fig. 1c). Furthermore, the protein levels of LRRK2 were increased by IFNγ exposure and this was accompanied by increased phosphorylatin of LRRK2 target, RAB10^11,12^ in IFNγ stimulated f-PD1 iMGL (Fig. 1d., Supp. Fig. 1c,d. Supp. Information).

Pairwise comparison identified 169 differentially expressed genes (DEGs) between the LRRK2-G2019S carrying iMGL (f-PD1 iMGL) compared to isogenic controls (f-ISO1 iMGL) at the basal level (Veh). IFNγ stimulation resulted in an additional 95 DEGs between f-PD1 and f-ISO1 iMGL. IFNγ stimulation led to 7214 and 7109 DEGs in f-ISO1 and f-PD1 iMGL, respectively, compared to basal conditions (Supp. Table S1). Functional enrichment analysis of the DEGs revealed that LRRK2-G2019S carrying iMGL significantly deregulated pathways linked to immune cell activation, cytokine storm, neuroinflammation signalling and metabolism (Fig. 1e, Supp. Table S2). Further IFNγ stimulation induced deregulations in pathways associated with cellular metabolism (Fig. 1e, Supp. Table S2) suggesting that LRRK2-G2019 alters microglial inflammatory responses and cellular metabolism.

To compare the transcriptional landscape of iMGL with in vivo human brain microglia, we integrated our dataset with published single-nuclei sequencing (snRNA-seq) data from human PD midbrain^30^. A deconvolution analysis of the snRNA-seq dataset confirmed that 80% of the microglia in the snRNA-seq dataset overlapped with Abud’s et al. microglia, and 75% with our microglia dataset (Fig. 1f), respectively. This re-confirmed the quality and microglial identity of our samples and demonstrated that all the three sequencing studies captured a similar type of microglia.

We then performed a functional analysis with the DEGs between the PD and the control microglia transcriptomes obtained from both our bulk RNA-seq of iMGL and the snRNA-seq data. We found that PD-microglia showed deregulation of neuroinflammation signalling, cytokine and immune cell activation pathways in both of the studies (Fig. 1g, Supp. Fig. 1e, Supp. Table S3) suggesting a PD-related alterations in the microglial transcriptome. For example, pathway analysis proposed activation of neuroinflammation signalling pathway in PD-microglia (Fig. 1h). We denominated the set of significantly altered pathways as the consensus transcriptional signature of PD-microglia, characterizing the commonalities between microglia in idiopathic PD brain and LRRK2-G2019S iMGL.

### Mitochondrial respiration is altered in LRRK2-G2019S carrying iMGL

Since the transcriptional PD-microglia signature suggested changes in immune and neuroinflammation related pathways, we wanted to investigate how this translates to iMGL functionality. For the functional studies, we included additional iPSC lines originating from healthy males (m-CTRL) and male PD-patients carrying the LRRK2-G2019S mutation (m-PD)^31,32^ in addition to a female isogenic pair (Table 1). iMGL differentiated from these iPSC showed expression of microglial-specific genes, such as *C1QA, GAS6, MERTK,* and *LRRK2* as detected by qPCR (Supp. Fig. 2a). In addition, all iMGL genotypes stained positive for microglial markers IBA1, P2RY12, TMEM119 and PU.1 (Supp. Fig. 2b). On average, over 40% of the iMGL responded to the application of nucleotides (ATP or ADP) with intracellular Ca^2+^ transient measured with Fluo-4 (Supp. Fig. 2c–e). m-PD iMGL showed a higher percentage of responsive cells compared to m-CTRL iMGL with both nucleotides. In contrast, the f-PD1 responses did not differ from the isogenic control. The amplitude of the nucleotide-evoked Ca^2+^ transient did not differ between PD and control iMGL (Supp. Fig. 1e).

**Figure 2:**
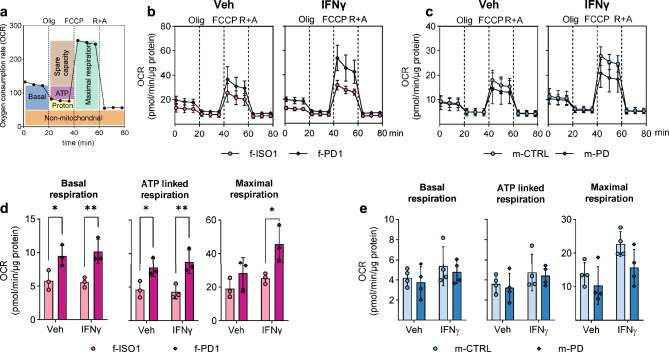
Mitochondrial respiration in LRRK2-G2019S carrying iMGL. Mitochondrial respiration of iMGL was measured with Seahorse Mito Stress test. (**a**) Schematic for the assay. Oxygen consumption rate (OCR) of iMGL was measured in response to sequential injections of oligomycin (Olig), carbonyl cyanide 4-(trifluoromethoxy)phenylhydrazone (FCCP) and a combination of rotenone and antimycin A (R + A) (**b**) in female isogenic iMGL pair and (**c**) in male iMGL pair and–CTRL and m-PD at the basal level and after IFNγ stimulation. (**d**,**e**) Mitochondrial parameters calculated from changes in the OCR. n = 3 batches (f-ISO1 and f-PD1) and n = 4 batches (m-CTRL and m-PD), with at least 7 technical replicates (wells) per group in both. Data are presented as mean ± SD. Two-way Anova with Sidak´s multiple comparison test, *p < 0.05, **p < 0.01. Any wells with negative OCR readings were considered outliers and excluded from the analysis.

Seahorse Mito Stress assay for the evaluation of mitochondrial respiration revealed a higher oxygen consumption rate (OCR) for iMGL carrying LRRK2-G2019S compared to the isogenic control resulting in higher and ATP-linked respiration at the basal state (Fig. 2a,b,d, Supp. Fig. 3a,c). Pre-stimulation with IFNγ resulted in higher basal, ATP-linked, as well as maximal respiration in f-PD1 iMGL. In contrast, no differences in the mitochondrial parameters were observed between m-CTRL and m-PD (Fig. 2c,e, Supp. Fig. 3b, d).

Rhodamine 123-dye (Rho123) based imaging of mitochondrial membrane potential (MPP) showed no differences in the basal fluorescence or carbonyl cyanide 4-(trifluoromethoxy)phenylhydrazone (FCCP) evoked response between PD-iMGL and control or isogenic line (Supp. Fig. 3e,f). Pre-stimulation with IFNγ resulted in an increased FCCP-response in the f-ISO1 and f-PD1 pair, but LRRK2-G2019S did not alter the responses (Supp. Fig. 3 g,h), indicating that MPP is not disrupted by this mutation.

### Nucleotide induced chemokinesis and migration are not altered in PD-iMGL

**Figure 3:**
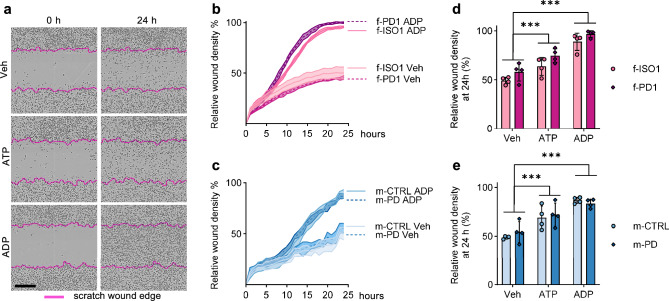
Effect of LRRK2-G2019S on iMGL chemokinesis. The chemokinesis of iMGL was measured using a live cell imaging system and scratch wound assay. (**a**) Representative phase images of iMGL chemokinesis after wounding a monolayer of iMGL (0 h and 24 h timepoints). Chemokinesis was observed in the presence of migration-inducing molecules ATP and ADP. Purple lines are used to mark the initial wound edge, black scale bar = 350 µm. (**b**, **c**) Representative (1 batch of iMGL) curves for the chemokinesis of the iMGL. The chemokinesis was measured using relative wound density, which measures the density of the cells inside the wound region related to the density of the cells region outside the wound region. n = 1 batch of iMGL, with 4–5 technical replicates (wells) per group, presented as mean ± SD. (**d**, **e**) Relative wound density at 24 h timepoint, measured from 4 independent batches (n = 4), with 4–5 technical replicates (wells) per batch for each group. Data are presented as mean ± SD. Two-way Anova with Sidak multiple comparison test was used for statistical testing. ***p < 0.001.

Real-time scratch wound assay/chemokinesis was used to elucidate the impact of LRRK2-G2019S in iMGL migration in the presence of microglial chemotactic molecules. A monolayer of iMGL was wounded and closure of the wound was observed for 24 h in the presence of 100 µM ATP or ADP (Fig. 3a) and migration was assessed by measuring the relative wound density (RWD) over the assay time. As expected, stimulation with ATP and ADP increased the iMGL migration and motility (Fig. 3b–e). The LRRK2-G2019S mutation did not alter these parameters in any studied conditions (Fig. 3d,e).

### Male PD-iMGL show dampened phagocytosis and increased cytokine secretion in response to inflammatory stimuli, but the effect is not replicated in female PD-iMGL

**Figure 4:**
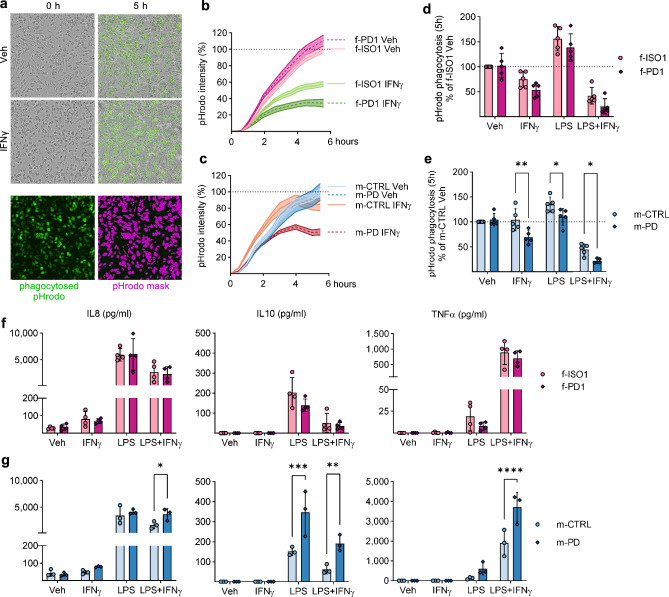
Effect of LRRK2-G2019S on phagocytosis and cytokines secretion in stimulated iMGL. The phagocytosis of iMGL was assessed using fluorescent pHrodo beads and Incucyte S3 Live-Cell Analysis System. (**a**) Representative images of the pHrodo phagocytosis and fluorescence intensity after (0 h and 5 h timepoints) in vehicle and IFNγ stimulated iMGLs. The phagocytosis was analyzed by measuring green fluorescence intensity. Purple color represents the pHrodo mask in the analysis. (**b**, **c**) Representative iMGL phagocytosis curves measured by green fluorescence intensity at the basal level and in IFNγ stimulated in iMGL. n = 1 batch, with 5 technical replicates (wells), presented as mean ± SD. (**d**, **e**) The phagocytosis of iMGL measured from pHrodo intensity at 5 h timepoint after stimulation with IFNγ, LPS or a combination of LPS + IFNγ. The results were normalized to vehicle treated control line (f-ISO1 or m-CTRL = 100%) for each batch of iMGL. n = 5 independent batches, with 5 technical replicates (wells) in each batch. The secretion of cytokines from iMGL in response to stimulation with IFNγ, LPS or LPS + IFNγ was measured from medium samples measured with cytokine bead array. (**f**) Secretion of IL8, IL10 and TNFα in female iMGL and (**g**) male iMGL. The cytokines were analyzed from medium samples collected from 4 (f-PD1, f-ISO1) or 3 (m-PD, m-CTRL) independent batches, with 5 technical replicates (wells). Data are presented as mean ± SD. Two-way Anova with Sidak ´s multiple comparison test, *p < 0.05, **p < 0.01, ***p < 0.001, ****p < 0.0001.

The impact of LRKK2-G2019S on the phagocytic capacity of the iMGLs was evaluated using pH-sensitive fluorescent beads under real-time live cell imaging in basal conditions and upon inflammatory stimuli (IFNγ, LPS or LPS + IFNγ; Fig. 4a–c). After 5 h, the development of fluorescence had reached a plateau and we used that timepoint to analyze the pHrodo intensity reflecting the phagocytosis (Fig. 4d,e). At basal conditions, LRRK2-G2019S did not alter the phagocytic capacity of the cells. Stimulation with IFNγ dampened phagocytosis in m-PD compared to m-CTLR, but the effect was not evident in the isogenic pair. LPS stimulation alone or in combination with IFNγ (LPS + IFNγ) resulted in dampened phagocytosis in m-PD compared to the control line in a similar manner. Still, no significant differences were seen in the isogenic pair.

To study how the LRRK2 mutation influences iMGL cytokine release in response to 24 h inflammatory stimuli, we measured cytokines from medium samples using a flow cytometry-based cytokine bead array (CBA). Whereas we detected no significant differences in cytokine secretion in the isogenic pair of iMGL (Fig. 4f), LRRK2-G2019S-carrying m-PD iMGL showed higher secretion of pro-inflammatory IL8 and TNFα and anti-inflammatory IL10 in response to LPS + IFNγ stimulation (Fig. 4g), suggesting altered cytokine release in response to heavy inflammatory stimulation in male PD-iMGL.

### Single-cell level description of PD signature microglial expression profile in human midbrain post-mortem samples

**Figure 5:**
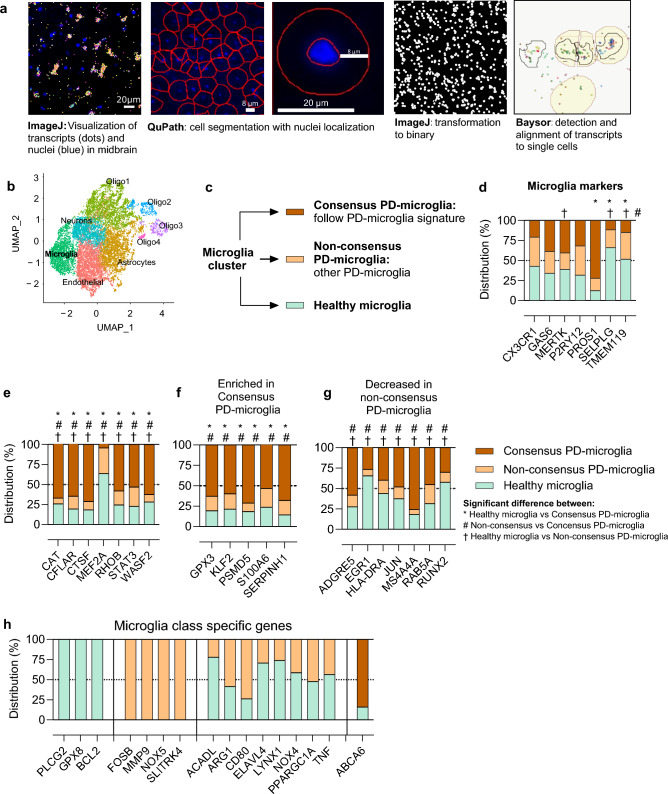
Gene expression profile of PD-microglia in human midbrain post-mortem samples. The human midbrain microglial expression of 100-plex gene panel was studied with Molecular Cartography from healthy and PD-patient midbrain samples. (**a**) Detected transcripts can be visualized with ImageJ and the single-cell analysis was carried out with automatic sell segmentation and assignment of transcripts to cells. (**b**) Visualization of annotated cell types in uniform Manifold Approximation and Projection for Dimension Reduction (UMAP) form. (**c**, **d**) Expression of microglial markers in the microglia cluster between different microglial classes presented as distribution percentage of average expression. Expression of genes which were (**e**) differentially expressed between all three classes, (**f**) enriched in consensus PD-microglia and (**g**) decreased expression in non-consensus PD-microglia. (**h**) Genes that were microglia class-specific. For the midbrain samples, n = 8 controls, n = 7 PD patients, one section per sample. Data presented as distribution percentage of average expression between the microglial classes, differential expression analyzed with the Wilcoxon rank sum test.

To study the PD microglial profile further, we employed the Molecular Cartography spatial transcriptomics method (Resolve Bioscience) to detect one hundred low level transcripts in the human PD midbrain post-mortem samples (Fig. 5a). We found and annotated five cell type-specific populations with unsupervised clustering (microglia, endothelial cells, neurons, astrocytes, and oligodendrocytes) (Fig. 5b). Cells of both PD and healthy donors populated each cell type in even proportions (Supp. Fig. 5b). The microglial population was defined by their high expression of microglial markers, including *TMEM119, P2RY12* and *MERTK*.

To study the midbrain PD-microglial states more closely, we utilized the transcriptional signature of PD-microglia defined earlier in this study (Fig. 1f). This PD-microglia signature consists of the overlap between the in vitro PD-iMGL and the human midbrain PD-microglia characterized by snSEQ dataset (Fig. 1f). Microglia detected with the spatial transcriptomics technique from the PD midbrain samples were classified into “consensus PD-microglia” and “non-consensus PD-microglia” (Fig. 5c). Compared to healthy microglia, consensus PD-microglia showed similar deregulation of transcripts as in PD-microglia signature (Fig. 1f). In contrast, non-consensus PD-microglia showed a different transcriptional profile compared to PD-microglia signature.

Consensus PD-microglia comprised of 5.4% (35 out of 653) of PD-brain microglia and revealed a unique expression profile. These microglia expressed the microglial marker *PROS1* more than other microglial cells, while it had an opposite expression pattern for *SELPLG* and *TMEM119,* which were significantly lower (Fig. 5d, Supp. Table S4). Genes with average expression significantly different between all three groups included *CAT, CFLAR, CTSF, MEF2A, RHOB, STAT2, WASF2* (Fig. 5e). All these, except *MEF2A*, were enriched in the consensus PD-microglia. *CAT* and *CTSF*, encoding for catalase and Cathepsin F respectively, were also expressed at a higher level in f-PD1 iMGL compared to their isogenic control (Supp. Table S1). *MEF2A* is characterized as a marker for homeostatic microglia, suggesting that the inflammatory state of consensus PD-microglia is altered. In addition, consensus PD-microglia differed from the other two types by higher expression of genes including *PSMD5* and *S100A6* (Fig. 5f). In iMGL, *PSMD5*, coding for a proteasome subunit, was significantly higher expressed in f-PD1 compared to their isogenic controls, but *S100A6*, coding for calcium binding protein, was downregulated in the mutation carrying iMGL. Non-concensus PD-microglia showed decreased expression of genes such as *HLA-DRA (*Major Histocompatibility Complex, Class II, DR Alpha) and *MS4A4A (*Membrane-spanning 4-domains subfamily A member 4A) (Fig. 5g).

Some genes were not expressed in all the microglial classes. For example, *PLCG2* (Phospholipase C Gamma 2), *GPX8* (Glutathione Peroxidase 8) and *BCL2* (BCL2 Apoptosis Regulator) were expressed only in healthy brain microglia. *FOSB* (AP-1 Transcription Factor Subunit), *MMP9* (matrix metallopeptidase 9), *NOX5* (NADPH Oxidase 5) and *SLITRK4* (SLIT And NTRK Like Family Member 4, involved in synaptogenesis) were solely expressed in non-consensus PD-microglia. (Fig. 5h). *ACADL* (Acyl-CoA Dehydrogenase Long Chain, mitochondrial flavoenzyme), *ARG1* (Arginase 1), *CD80, ELAVL4* (ELAV Like RNA Binding Protein 4), *LYNX1* (Ly6/Neurotoxin 1), *NOX4* (NADPH Oxidase 4), *PPARGC1A* (PPARG Coactivator 1 Alpha), *TNF* (Tumor Necrosis Factor) were absent from consensus PD-microglia and *ABCA6* (ATP Binding Cassette Subfamily A Member 6) was not expressed in non-consensus PD-microglia.

## Discussion

Here we show that iMGL carrying LRRK2-G2019S recapitulate a part of the transcriptional signature of human PD brain microglia. The altered pathways were related to phagocytosis, lysosomal functions and oxidative stress and translated into deficits in iMGL phagocytosis, cytokine production and mitochondrial functions. In addition, we describe transcriptional alterations in LRRK2-G2019S carrying microglia and how these alterations correspond to human PD brain microglia.

A shared neurodegenerative microglial transcriptional signature has been described in the mouse models of amyotrophic lateral sclerosis, multiple sclerosis, and AD^33^. However, in PD, disease-associated microglial states are less well characterized. Single-cell sequencing of mouse brain revealed that microglia show different activation states between striatum and midbrain. Midbrain microglia were defined as being “immune alert”, characterized by increased expression of inflammatory and antigen presenting markers and lower level of microglia homeostatic genes^34^. In contrast, midbrain microglia in an aSyn overexpressing mouse model of PD show anti-inflammatory signature compared to a pro-inflammatory state in striatal microglia^35^. In the human brain, a single cell laser-capture microscopy-based study showed that PD-related alterations in the microglial transcripts did not associate with inflammation, but rather pathways related to neuronal repair^36^. Yet, a snRNA-seq of microglia in the midbrain highlighted an inflammatory shift and an alteration in unfolded protein response in PD patients^30^. Interestingly, AD and PD patient microglia from disease-specific brain sites share only very few overlapping transcripts^36^. Thus, transcriptional investigation of midbrain microglia is essential in the context of PD.

Our mRNA-multiplex data from human midbrain revealed that microglia in PD brain have different states. A small population (around 5%) followed the PD-microglia signature defined by bulk RNAseq from iMGL and snRNA-seq of microglia from human midbrain, which we defined as consensus PD microglia signature, indicating a shared transcriptional fingerprint between the models and our samples. The loss of homeostatic microglial genes (*CX3CR1*, *P2RY12, TMEM119)* can be a marker for a disease state in other neurodegenerative diseases^33,37^. The consensus PD-microglia showed some level of similarity with common disease-associated microglia in the loss of homeostatic genes, such as *TMEM119, SELPG, MEF2A*, but on the other hand, the expression of *CX3CR1* or *P2RY12* was not altered. The enrichment of genes related to phagocytosis (e.g. *PROS1, WASF2*), lysosomal degradation, oxidative stress (e.g. *CAT, CTSF*) and proteasome function (*PSMD5*) in the consensus microglia may suggest responses to the pathological environment in the PD brain, and possible alterations in the microglial phagocytic and lysosomal machinery. This conferred as dampened phagocytosis and increased cytokine secretion in our LRRK2-G2019S carrying iMGL. Prior studies have linked the expression of these genes to PD pathology. *WASF2* (also known as *WAVE2*) is known to regulate myeloid cell phagocytosis in PD^38^. Enrichment in antioxidant gene expression, including catalase, has been reported in aSYN-overexpressing microglia^39^. Different cathepsins (proteases, involved in lysosomal proteolytic system) are involved in the lysosomal clearance of aSYN^40^. Proteosomes are an important part of the degradation of aSYN and alterations in the subunits and proteasome compositions have been reported in PD^41^. Monocytes extracted from PD-patients show deregulation in the endo-lysosomal pathways, and a subpopulation of monocytes show impairment in the membrane structures as a possible manifestations of gene level deregulation^42^. Another study found deregulation of mitochondrial and proteasomal pathways which were enriched in the intermediate monocyte population^43^. These studies highlight the importance of lysosomal and proteasomal pathways in PD and support our findings in LRRK2-G2019S carrying iMGL microglia.

In contrast to consensus PD-microglia, non-consensus PD-microglia reduced expression of many genes present in its consensus counterparts. In addition, *MERTK,* which is an important player in microglial phagocytosis^44^, was reduced in the non-consensus PD-microglia. The non-consensus PD-microglia expressed some genes with lower expression in consensus PD-microglia, such as *NOX4, NOX5, ACADL. NOXs* encode for different NADPH oxidases and have been implicated in PD by reactive oxygen species production^45^.

At the functional level, LRRK2-G2019S caused alternating changes in iMGL. Female PD-iMGL were characterized by altered mitochondrial respiration compared to isogenic control, whereas male PD-iMGL showed dampened phagocytosis and increased cytokine expression compared to healthy controls. Prior studies utilizing human-derived cellular models have reported somewhat contradictory findings on the impact of LRRK2-G2019S on cellular functions. Human iPSC-derived LRRK2-mutant monocytes show reduced migration at the basal state compared to non-mutation carrying controls^46^. On the contrary, Panagiotakopoulou et al. (2020) reported increased migration and phagocytosis and altered cytokine secretion and metabolic profiles in iPSC-derived microglia carrying LRRK2-G2019S^21^. iPSC-derived microglia from idiopathic PD patients were also characterized with increased phagocytosis and expression of the *IL1B* and *IL10* genes^47^. These discrepancies might be caused using different cellular models to study microglia and different protocols to generate them. Our iMGL are cultured in the presence of FBS and it has been argued that factors in serum may prime microglial function^48^, possibly recapitulating aspects of the more immune-alert state of midbrain microglia^34^ In addition, the sex of the patient donors is likely to influence microglial functions. PD is more common in males compared to females^49^ and differences between male and female patients have been reported in different aspects of PD, such as males presenting a more rapid decline in cognitive impairment and deficits in smell compared to females^50–52^. In addition, sex has been shown to have a role in rodent microglia; for example, male rodent cortical microglia are more responsive to ATP and express higher levels of antigen-presenting molecules (MHCI and MHCII) as well as purinergic receptors (P2RX7, P2RX4, and P2RY12)^53^.

Caveats in our study should also be noted. We investigated the LRRK2-G2019S mutation only, and with one isogenic pair of iPSC. To increase the number of lines in our study, we also included additional LRRK2-G2019 mutant male lines (Table 1). Due to individual, line-specific differences in the functional readouts, our study should be repeated with additional lines including male isogenic pairs to draw more definitive conclusions on how LRRK2-G2019S impacts iMGL function. Unfortunately, we are not able to draw any sex-specific conclusions, as this would in fact require a large set of both healthy control and LRRK2 mutation carrying lines. The microglia we generated were cultured for a relatively short time and as a 2D culture. These cells lack a mature microglial identity due to the lack of 3D environment, limited maturation time in culture and the environment in which microglia interact with other cell types such as neurons and astrocytes. This leads to lack of extensive microglia-like morphological features, such as ramifications, observed in microglia in vivo or in 3D culture. Despite these inherent differences due to the 2D culture conditions, our LRRK2-mutation carrying iMGLs were able to recapitulate some aspects of a PD-specific microglial signature at the transcriptional level, pinpointing that the model is relevant for investigation of specific aspects of microglial biology in PD.

To conclude, our study suggests that LRRK2-G2019S causes subtle and line-dependent alterations in the functionality of iPSC-derived microglia. As the G2019S mutation in LRRK2 is the most common cause of late onset PD, it serves as good platform to investigate the effects of PD-associated genetic defects on the microglial fitness and behavior, especially because of its association with immunity and microglia. Our iMGL model resembles human microglia and shows suitability to study PD-associated mutations in microglia.

## Material and methods

### Acquisition, generation, and maintenance of iPSC

The female iPSC-lines (f-PD1, f-ISO1) were previously generated and characterized by (Reinhardt et al., 2013), with the consent approved by The Ethics Committee of the Medical Faculty and the University Hospital Tübingen. The reprogramming of patient sample resulting in the line m-PD (CSC-22A) was regulated by a permit delivered to Dr. Laurent Roybon by the Swedish work environment authority and registered under the number 2020-3211 and the iPSC line has been previously characterized^32^. Characterization of m-CTRL line is described in^31^ under the ethical permission Northern Savo Hospital district (license no. 123/2016). Lines are listed in the Table 1. Informed consent was obtained from all the donors. All experiments with iPSCs and iPSC-derived cells were performed in accordance with the Declaration of Helsinki, with the permission from the Research Ethics Committee of the Northern Savo Hospital District (license no. 123/2016).

**Table 1:** iPSC-lines used in this study.

Alias	Alternative name	Age at biopsy	Sex	Health status	Mutation genotype	Karyotype	References
f-PD1	LRRK2 Patient #1	52–53 y	F	PD	LRRK2-G2019S	46, XX*	^54^ (*this manuscript)
f-ISO1	LRRK2 Patient #1	52–53 y	F	isogenic for f-PD1	LRRK2 wildtype	46, XX*	^54^ (*this manuscript)
m-CTRL	MAD6	63 y	M	Healthy	LRRK2 wildtype	46, XY	^31^
m-PD	CSC-22A	57 y	M	PD	LRRK2-G2019S	46, XY	^32^

The chromosomal stability of f-PD1 and f-ISO1 iPSC was studied by G-banding and 300-band resolution. iPSC-lines were arrested to metaphase using 200 ng/ml KaryoMAX™ Colcemid™ (Gibco). 20 metaphases were analyzed at the Eastern Finland Laboratory Centre Joint Authority Enterprise (ISLAB, Kuopio, Finland). All iPSCs were maintained in complete Essential 8™ medium (Gibco) supplemented with 50 units/ml of Penicillin and 50 µg/ml Streptomycin (0.5% P/S, Gibco) on Matrigel (1:200 dilution in DMEM/F-12, Corning) 5% CO_2_, + 37 °C. The cells were passaged using 0.5 mM EDTA (Invitrogen), in the presence of 5 µM ROCK1 inhibitor Y-27632 (Selleckchem) every 3–4 days. The iPSCs were confirmed to be negative for mycoplasma using the MycoAlert Kit (Lonza).

### Differentiation of microglial-like cells (iMGL)

Microglial-like cells (iMGL) were differentiated from iPSCs by following our previously published protocol^55^ with minor differences. Briefly: for the first 4 days, iPSC are differentiated in hypoxia (5% O_2_). On differentiation day 0 (D0), the seeding density of iPSC varied from 7500–15,000 single cells/cm^2^. To guide iPSC towards mesodermal lineage, E8-medium was supplemented with 5 ng/ml BMP4, 25 ng/ml Activin A (both Peprotech), 1 µM CHIR99021 (Axon) on D0 and D1. ROCK1 inhibitor (Selleckchem) was used to inhibit the cell death on first two days (D0: 10 µM, D1: 1 µM). On D2, medium was changed to home-made base medium: DMEM/F-12 supplemented with 1X GlutaMAX, 0.5% P/S, 543 mg/l sodium bicarbonate (all Gibco), 64 mg/l L-ascorbic acid and 14 μg/l sodium selenite (both Sigma). This base medium was supplemented with 100 ng/ml FGF-basic, 50 ng/ml VEGF (both Peprotech) 10 μM SB431542 (Selleckchem), 5 μg/ml insulin (Sigma) on D2 and D3. On D4, base medium was supplemented with 50 ng/ml FGF-basic, 50 ng/ml VEGF, 50 ng/ml TPO, 10 ng/ml SCF, 50 ng/ml IL6 and 10 ng/ml IL3 (all Peprotech) and 5 μg/ml insulin (Sigma) and cultured in normoxia with daily media changes until D8. On D8 onwards, the floating progenitor cells were collected and maintained in microglial base medium containing Iscove’s modified Dulbecco’s medium (IMDM, Gibco), 0.5% P/S, and 10% heat inactivated fetal bovine serum (FBS, Gibco) supplemented with 5 mg/ml insulin (Sigma) 5 ng/mL MCSF and 100 ng/mL IL-34 (both PeproTech) on ultra-low attachment dishes (Corning). On D10 onwards, the microglial base medium was supplemented with 10 ng/ml of both, MCSF and IL-34. On D16, cells were seeded on experiment plates and matured until experiment. Half of medium was changed daily.

For Western blot analysis, iMGL differentiated from frozen progenitor (D8 state) cells. Briefly: D8 progenitors were frozen with IFP supplemented with 10% DMSO and stored in liquid N2. Progenitors were thawed and plated on ULA-dishes (5 million per dish) in MP-medium with 5 µM of ROCK inhibitor. On the next day, medium was changed without ROCK inhibitor and the culture was maintained as above.

### RNA-sequencing and analysis

iMGL were seeded in the density of two million cells per 6 cm dish and the total RNA was extracted using mirVana™ miRNA Isolation Kit (Invitrogen) according to the manufacturer´s instructions. The samples were purified using Zymo RNA clean and concentrator-5 (Zymo research) and quantified with Qubit 2.0 Fluorometer (Invitrogen, LifeTechnologies). The integrity of RNA samples was measured with RNA 6000 Nano kit and Bioanalyzer 2100 (Agilent).

Libraries were prepared with Corall Total RNA-seq library preparation kit combined with RiboCop (Lexogen) for removal of ribosomal RNA. For indexing, the Lexogen i5 6 nt Unique dual indexing Add-on Kit was used, and the optimal cycle amount was assessed with PCR add-on kit (Lexogen). The quality of prepared cDNA libraries was confirmed with the High Sensitivity DNA Analysis kit (Agilent). Sequencing was carried out at Genome center of Eastern Finland (UEF) with Illumina NextSeq500 sequencer using Illumina NextSeq 500/550 High Output Kit v2.5, 75 cycles -kit with NextSeq PhiX Control Kit (both Illumina).

### RNA-seq data analysis

Reads were aligned and quantified to the human reference genome GRCh38 using the nf-core workflow^56^ (3.8.1 version of “rnaseq”). Count data were prepared following the workflow defined by Law et al.^57^. Lowly expressed molecules were filtered out with the “filterByExpr” function with default parameters to increase the reliability of the mean–variance relationship. Differences between samples due to the sequencing depth were removed by normalizing the count using the trimmed mean of M-values (TMM)^58^ method and applied a log transformation minimizing sum of sample-specific squared difference to enhance the true positive and negative ratio in the downstream analysis^59^. Outliers and sample features were checked by performing an unsupervised consensus clustering with Cola^60^. The results showed a clear separation of the groups, and no outlier gene or sample was detected. Quality control was finalized by checking how much cell type-specific markers were enriched and coordinatively expressed in the samples. Cell type specific data was collected from PanglaoDB^61^ and determined how much each set of markers was enriched with the ssGSEA score^62^. A design matrix for each pair of conditions to compare was created and differential expression analysis was performed using the limma/edgeR model^57^ controlling for the false discovery rate with Benjamini–Hochberg Procedure^63^. In addition, a similar analysis was performed also for Abud et al.^29^ RNA sequencing data. Once both data sets were merged, correlation analysis was performed to determine the similarity with the Spearman correlation^64^. Differentially expressed genes were uploaded to QIAGEN IPA (QIAGEN Inc., https://digitalinsights.qiagen.com/IPA)^65^ for Ingenuity Pathway Analysis (IPA) and functional enrichment analysis. The analysis has been performed with default parameters with the human annotation.

### snRNA-seq data analysis and deconvolution of RNAseq data

To compare the iMGL bulk sequencing data to brain microglia, the data from previously published single-nuclei RNA sequencing (snSEQ) from human midbrain^30^ including midbrain samples from PD patients and matched controls was collected. Data was prepared and analyzed with Seurat^66^. The cells annotated by the original authors were kept and the data was normalized following the “Standard pre-processing workflow”. The principal component analysis was performed to reduce the dimensionality of the dataset with the author’s most variable genes to confirm that similar results were obtained. Finally, differentially expressed genes between PD and control samples were determined with the Wilcoxon rank sum test.

To determine, which midbrain cell types were determining our iMGL expression profile captured by bulk RNAseq, the annotated and prepared count matrix of Smajić et al. was provided to MuSiC^67^. MuSiC software is designed to work with multi-subject single-cell level sequencing dataset, and this deconvolution results to a percentage for each cell type in the snRNAseq. The percentage indicates how much a cell type contributes to the expression profile of sequenced sample. For the radar chart, the percentages of each sample’s class are equal to the average per cell type of the percentages of its members and hence, the sum of the percentages is not equal to 100.

To define the shared PD-microglia signature between midbrain microglia and our iMGL, the differentially expressed genes (PD versus control microglia & f-PD1 versus f-ISO1 iMGL) were provided as a input to IPA for performing a pathway analysis. Significantly deregulated pathways, which did not include genes from both datasets were filtered out, defined the PD-microglia signature.

### Spatial detection of transcripts in human brain with Molecular Cartography™ technology

Queen Square Brain Bank (QSBB) tissue is collected under ethical approval from the NHS research ethics committee (NEC) and in accordance with the human tissue authority's (HTA's) code of practice and standards under license number 12198, and tissue is used under the approval of the Regional committees for medical and health research ethics (REK, Norway), license number 2018/124. The specific information provided by the samples is listed in the Table 2. The frozen brain samples were cryo-sectioned and a 10 µm thick piece of each sample was placed on the Molecular Cartography imaging glass slide and the samples were shipped to Resolve Biosciences for analysis. The expression of 100 transcripts (Supplementary Information) in the tissue samples was studied using Molecular Cartography, which is based on fluorescent in situ hybridization of single molecules with multiplexing. Regions of interest for each sample were selected from phase images according to tissue quality. Areas with any folded tissue was not selected. In addition, any big holes or tears in tissue were avoided if possible. More detailed information of sample preparation, Molecular Cartography analysis at Resolve Biosciences and list of probes is described in the Supplementary Information.

**Table 2:** Details of the brain samples used for the spatial analysis.

Patient No	Age	Sex	PMD	PD Braak	AD Braak
PD cases					
PD1	80	F	37,10	5	II
PD2	78	M	54,55	5	II
PD3	79	F	46,20	6	I
PD4	89	M	39,00	6	II
PD5	83	F	41,00	6	II
PD6	81	M	38,00	6	II
PD7	83	M	29,45	6	II
Controls					
CTRL1	86	F	40,20	N/A	N/A
CTRL2	87	F	51,45	N/A	I
CTRL3	89	M	38,30	N/A	III
CTRL4	83	M	63,30	N/A	III
CTRL5	76	M	79,00	N/A	II
CTRL6	92	F	86,50	N/A	III
CTRL7	91	F	71,40	N/A	II
CTRL8	96	F	36,00	N/A	II

PMD = post mortem delay (hours). PD Braak defined by score for Lewy bodies (a-synuclein staining). Alzheimer’s disease (AD) Braak stage defined by Amyloid-β staining, no pathological diagnosis for neurodegenerative diseases. N/A: not available.

The transcript expression coordinates and the corresponding DAPI stained image of the tissue were used for the analysis. For the single-cell level analysis, cell segmentation was done with QuPath (version 0.4.0)^68^ and ImageJ (version 1.53S)^69^. In QuPath, following settings were used: image type: fluorescence, pixel width: 0.138 µm, pixel size: 0.25, background radius: 8, sigma: 1.7, threshold: 25 and cell expansion: 8 (without including the cell nucleus). In ImageJ, the image was set up as 8-bit, the colors were flattened, threshold function was used to increase the contrast and the results were saved as binary mask. The mask and transcript coordinates were transported to Baysor (version 0.5.2)^70^ for assignment of transcripts into segmented cells. The software output is a count matrix describing each segmented cell with an expression profile resulting in multiple count matrices. The final count matrices were then analyzed with Seurat (version 4.8)^66^ following the “Standard pre-processing workflow”. However, due to nature of the analysis method, no cells were filtered out based on their number of transcripts and we selected only the 10 most variable transcripts. The principal component analysis was performed, and the cells were clustered according to the five components explaining the most of their variablity. The clusters of cells have been manually annotated with the cell type-specific markers selected for the experiment.

We noticed that *GFAP* contributed 49–89% of detected transcript numbers and was evenly spread around the tissue (Supp. Figure 4a.) Hence, to avoid skewing the single-cell level analysis, *GFAP* was removed from the subsequent analysis.

Microglia from the PD-brain were classified as “consensus PD-microglia” if the gene expression profile followed the PD-microglia signature defined by our iMGL RNAseq and snSEQ^30^. Meaning that the DEGs were deregulated in the same manner as in the bulk and snSEQ. Other microglia from PD-brain were classified as “non-consensus microglia”. Average expression in between the groups was presented as distribution percentage per each gene. The differential expression was analyzed with the standard Wilcoxon rank sum test.

### iMGL functional assays

iMGL were seeded on poly-D-lysine (PDL)-coated experiment plates on D16 and half of the microglial medium was changed daily. All functional assays were performed between D22-24. Unless stated otherwise, iMGL were starved without FBS 24 h before assays and additionally, stimulated with 20 ng/ml LPS (serotype O111:B4, Sigma, L2630), 20 ng/ml IFNγ (PeproTech) or with a combination of both (LPS + IFNγ).

### Immunocytochemistry

For immunostaining, 30,000 iMGL were seeded on µ-Slide 8-Well plates (80826, Ibidi) iMGL were fixed with 4% PFA, permeabilized with 0.5% Tween-20, 0.2% Triton X-100 in 5% Normal goat serum (NGS) followed by blocking with 0.2% Triton in 10% NGS. Blocking was followed by overnight incubation at + 4 °C with primary antibodies (all produced in rabbit): 1:200 Anti-Iba1 (019–19741, Wako), 1:125 Anti-P2RY12 (HPA014518, Sigma-Aldrich), 1:100 Anti-Tmem119 (ab185333, Abcam) or 1:200 PU.1 (2266S, CST). Secondary antibody (Alexa Fluor 568, Invitrogen A11011, 1:500) was incubated overnight + 4 °C and nuclei were stained with bizbenzimide. A well without primary antibody was used as staining control. The iMGL were imaged with Zeiss AxioObserver microscope equipped with a LSM700 confocal module (Carl Zeiss MicroImaging GmbH, Jena) using the z-stack mode and 40X magnification. The z-stack images were combined in Zen Black software (Carl Zeiss MicroImaging GmbH) using maximum intensity projection for image presentation.

### RT-qPCR

To measure expression of microglia specific genes, iMGL were seeded in the density of 2 million cells per Poly-d-Lysine-coated (PDL, Sigma) 6 cm dishes (Sartorius). RNA was extracted with Trizol-reagent (Invitrogen) according to manufacturer´s instructions. The concentration of RNA was measured with Nanodrop 2000 spectrophotometer (Thermo Fisher Scientific, Hudson, NH). 500 ng of RNA was used for reverse transcription to cDNA with Random hexamer primers (Fermentas). The levels of mRNA were measured from the samples using the StepOne Plus Real-Time PCR system (Life Technologies) and TaqMan assay mixes (Thermo Fisher Scientific) with FAM-MGB dye for following targets: *AIF1* (Hs00610419_g1), *C1QA* (Hs00706358_s1), *GAS6* (Hs01090305_m1), *GPR34* (Hs00271105_s1), LRRK2 (Hs00968209_m1), *MERTK* (Hs01031979_m1), *PROS1* (Hs00165590_m1), *TREM2* (Hs00219132_m1). Results were normalized to housekeeping gene (*GAPDH,* Hs02758991_g1*).*

### Western blot

For protein extraction, 1 million iMGL were seeded on 6 cm dish. Proteins were extracted using in-house made RIPA buffer (50 mM Tris, 1% Triton-X 100, 0.5% Sodium deoxycholate, 0.1% SDS, 150 mM NaCl) with protease and phosphatase inhibitors (both Roche). Total protein concentrations were measured with Pierce™ BCA Protein Assay Kit (ThermoFisher Scientific, Waltham, MA, USA). Samples were mixed with Laemmli sample buffer (60 mM Tris, 10% glycerol, 2% SDS, 1% Bromophenol blue, with 5% 2-Mercaptoethanol added just before use). Samples were boiled for 5 min and 15 µg of protein was resolved with 12% (RAB10) and 4.5% (LRRK2) gels in Tris–Glycine running buffer.

Proteins were transferred to 0.2 µm PVDF pre-cut membrane transfer pack (1704157, Bio-rad) and transferred with Trans-Blot Turbo® transfer system (Bio-Rad, Hercules, CA, USA). Membranes were blocked in 5% BSA in TBST (20 mM Trish, 150 mM NaCl, 0.1% Tween-20, pH 7.6) and the membranes were incubated overnight at 4°C with primary antibodies diluted in blocking buffer (1:1000): Anti-LRRK2 [MJFF2 c(41-2)] (ab133474, Abcam) and Anti-Rab10 (phospho T73) [MJF-R21] (ab230261, Abcam). Membranes were washed and incubated with HRP-conjugated secondary antibody (Goat anti-rabbit, 1706515, Bio-rad) for 2 h in room temperature. After washing, decection was perfomed with Clarity™ Western ECL subtrate (1705060, Bio-rad) and ChemiDoc™ MP Imaging System (Bio-Rad, Hercules, CA, USA). For normalization, membranes were incubated with anti β-actin primary antibody (1:1000, A5441, Sigma) diluted in 5% BSA in TBST overnight at 4°C. Cy5-conjugated secondary antibody (donkey anti-mouse, 715-175-151, Jackson ImmunoResearch) was diluted in 1:1000 in TBST and incubated for 2 h at room temperature. After washing, the membrane was imaged with the same equipment as previously. The intensity of the target bands were quantified with ImageLab (version 5.1, Bio-Rad Laboratories) with autoscale settings.

### Ca^2+^-imaging and mitochondrial membrane potential

For studying the calcium influx and mitochondrial membrane potential, iMGL were seeded on PDL-coated glass coverslips. The ATP and ADP included calcium influx was studied as previously described^55^. Shortly: iMGL were loaded with the calcium-sensitive fluorescent dye Fluo-4AM (5 μM, Invitrogen) and iMGL response to short application of ATP or ADP was measured. The maximal response was evoked with ionomycin. For the analysis, the baseline subtracted maximum amplitudes of ATP or ADP responses were divided by ionomycin responses.

For the mitochondrial membrane potential measurement, iMGL were loaded with membrane-permeant green fluorescent dye Rhodamine 123 (5 µM, Rho123, Invitrogen) followed by washout as for the Ca2 + -imaging. Rho123-loaded cells were imaged with an excitation light wavelength of 495 nm (exposure time 50 ms, binning 2) and registered emission intensity ≥ 520 nm (exposure time 50 ms, binning 2). To test the functional state of mitochondria, the basic fluorescence was measured for 3 min, followed by application of 4 µM carbonyl cyanide 4-(trifluoromethoxy)phenylhydrazone (FCCP, Sigma) for 1 min. The data for Ca^2+^ and Rho123 imaging were pre-analyzed offline using the FEI offline analysis (TILL Photonics) and Image J, and further analysis was automatized using Origin2019 software (OriginLab).

### Mitochondrial respiration and energy phenotype (Seahorse XFe96 and MitoStress)

The mitochondrial metabolism of iMGL was measured with the Mito Stress assay (Agilent) according to manufacturer´s instructions. iMGL were seeded at the density of 60,000 cells/well on a Seahorse XF96-well plate from XFe96 FluxPak (102416-100, Agilent) according to the manufacturer’s instructions. On the day of the assay, XF assay medium (103575-100, Agilent) was supplemented with 25 mM glucose (Sigma), 2 mM l-glutamine and 1 mM sodium pyruvate (both Gibco). The changes in oxygen consumption rate (OCR) were measured using Seahorse XFe96 analyzer (Agilent) in response to sequential injections of 1.) Oligomycin 2.) Carbonyl cyanide 4-(trifluoromethoxy)phenylhydrazone (FCCP) 3.) Rotenone and Antimycin A (all Sigma), all reagents in the final concentration of 1 µM. The results were normalized to total protein amount measured with the Pierce™ BCA Protein Assay Kit (Thermo Scientific). The data was analyzed with XF Cell Mito Stress Test Report Generator (Agilent) according to the manufacturer´s instructions. Wells with negative OCR readings were considered outliers. Data calculated with the report generator was used to produce energy phenotype data based on the XF Energy phenotype test (Agilent). “Baseline” state were the OCR and the extracellular acidification rate (ECAR) from the last measurement before injection of oligomycin. “Stressed” state was the maximal OCR and ECAR rates after injection of FCCP. Data was presented as a graph with OCR/ECAR.

### Scratch wound assay

iMGL were seeded on IncuCyte® Imagelock (4379, EssenBioScience) plates in the density of 30,000 cells/well to obtain a confluent monolayer. For the whole duration of the assay, FBS was included in the medium. The cells were pre-stimulated with 100 µM ATP or ADP (both Sigma) for 2 h. The 96-well WoundMaker (Sartorius) was used to create wounds in the monolayer according to manufacturer´s instructions (Sartorius, IncuCyte® Scratch Wound Assay). The migration of iMGL in the presence of ATP or APD was imaged using Scratch wound scanning at least every 2 h for 24 h. The migration was analysed with IncuCyte 3S 2019B software. Wells with uneven wounds or unfocused imaging were excluded from analysis.

### Phagocytosis assay

iMGL were seeded on IncuCyte® ImageLock 96-well plate (4379, EssenBioScience) in the density of 15,000 cells/well. The pHrodo™ Green Zymosan Bioparticles™ (Invitrogen) for phagocytosis were diluted in Opti-MEM (Gibco) into a final concentration of 125 µg/ml. The pHrodo bioparticle solution was supplemented with the stimulations (LPS, IFNγ and LPS + IFNγ) and the bioparticles were added to the pre-stimulated iMGL. For negative control, well of iMGL in each treatment was treated with only OPTI-MEM. The iMGL were imaged with bright field and green fluorescent channels every 30 min, for 6 h using IncuCyte 3S live-cell analysis system. The images were analyzed by IncuCyte S3 2019B software. Masks for green fluorescence from phagocytosed bioparticles was quantified with Top-Hat segmentation. Threshold for excluding unspecific background fluorescence was set with negative control. Integrated green calibrated units was normalized to cell confluence. Wells with unfocused imaging were excluded from analysis.

### Cytokine secretion

The secretion of cytokines was detected from the medium samples of iMGL after 24 h stimulation with LPS, IFNγ or LPS + IFNγ. The concentrations of (hMCP1, hRANTES, hGM-CSF, hTNF, hIL-10, hIL-6, hIL-8) were analyzed using the Cytometric Bead Array (CBA) with human soluble protein flex sets (all from BD Biosciences). The samples and cytokine standards were incubated with the bead mixture (1:75 bead dilution) followed by incubation with the detection reagent (1:75 reagent dilution). Samples were run on Cytoflex S (Beckman Coulter) and at least 300 events for each cytokine were detected. Excitation was done with 638 nm red laser, and the bead clusters were detected with 660/20 BP (APC) and 780/60 BP filters (APC-A750). Cytokine reporter PE was excited with 561 nm yellow laser and filter 585/42 BP was used for detection. The data was analyzed with FCAP array (SoftFlow) to calculate cytokine concentrations from known standards.

### Statistical analysis

Outliers were detected with Grubb´s test, if not stated otherwise. Data was analyzed with GraphPad Prism 9 (GraphPad Software, San Diego, California), with Two-way Anova using Šidák multiple correction unless stated otherwise. P-values less than 0.05 were considered significant.

### Supplementary Information

Supplementary Table S1.

Supplementary Figure S1.

Supplementary Figure S2.

Supplementary Figure S3.

Supplementary Figure S4.

Supplementary Information 1.

Supplementary Table S2.

Supplementary Table S3.

Supplementary Table S4.

## Data Availability

The RNA sequencing and spatial transcriptomics data that support the findings of this study are available from the corresponding author upon reasonable request through Zenodo (link in Supplementary Information). In addition, the data used to compare in this study are available through GEO: GSE89189^29^ and GSE157783^30^.
