# Supplementary material for: Human iPSC-derived microglia carrying the LRRK2-G2019S mutation show a Parkinson’s disease related transcriptional profile and function
Source: Sci Rep. 2023 Dec 13;13:22118. doi: 10.1038/s41598-023-49294-9 (PMC10719377; doi:10.1038/s41598-023-49294-9)
Supplement: Supplementary file 1 — Supplementary Table S1. [file 41598_2023_49294_MOESM1_ESM.docx]

**Supplementary Information**

**Human iPSC-derived microglia carrying the LRRK2-G2019S mutation show a Parkinson’s disease related transcriptional profile and function**

**Short running title: LRRK2-G2019S and human microglia**

**Authors:** Sohvi Ohtonen^1*^, Luca Giudice^1^, Henna Jäntti^1a^, Mohammad Feroze Fazaludeen^1^, Anastasia Shakirzyanova^1^, Mireia Gómez-Budia^1^, Nelli-Noora Välimäki^1^, Jonna Niskanen^1^, Nea Korvenlaita^1^, Ilkka Fagerlund^1^, Jari Koistinaho^1,2^, Mahmood Amiry-Moghaddam^3^, Ekaterina Savchenko^4^, Laurent Roybon^4b^, Šárka Lehtonen^1,2^, Paula Korhonen^1^, Tarja Malm^1^

^1^ A.I. Virtanen Institute for Molecular Sciences, University of Eastern Finland, Finland

^2^ Neuroscience Center, University of Helsinki, Finland

^3^ Division of Anatomy, Department of Molecular Medicine, Institute of Basic Medical Sciences, University of Oslo, Norway

^4^ Stem Cell Laboratory for CNS Disease Modeling, Department of Experimental Medical Science, Lund University, Sweden

^a^  Current affiliation: F.M. Kirby Neurobiology Center, Boston Children's Hospital, Boston, MA, USA & Stanley Center for Psychiatric Research, Broad Institute of MIT and Harvard, Cambridge, MA, USA.

^b^ Current affiliation: Department of Neurodegenerative Science, the MiND program, Van Andel Institute, Grand Rapids, MI, USA.

*Corresponding author, correspondence to [sohvi.ohtonen@uef.fi](mailto:sohvi.ohtonen@uef.fi)

**Content**

Supplementary methods

Suplementary data

Supplementary full-sized Western blot

Supplementary figure legends

Supplementary table legends

**Supplementary methods**

**Spatial detection of transcripts in human midbrain with Molecular Cartography™ technology**

Fresh frozen post-mortem midbrain samples were sectioned with into 10 µm thick sections and placed within the capture areas of cold Resolve Biosciences slides. Samples were then sent to Resolve BioSciences on dry ice for analysis. Upon arrival, tissue sections were thawed and fixed with 4% v/v Formaldehyde (Sigma-Aldrich) in 1x PBS for 15 min at 4 °C. After fixation, sections were washed three times in 1x PBS for one minute, followed by one minute wash in 70% Ethanol and isopropanol at room temperature. Fixed samples were used for Molecular Cartography™ (100-plex combinatorial single-molecule fluorescence in-situ hybridization) according to the manufacturer’s instructions (protocol 1.3; available for registered users), starting with the aspiration of ethanol and the addition of 5% Trueblack followed by buffer DST1, tissue priming and hybridization. Briefly, tissues were primed for 30 minutes at 37°C followed by 24h hybridization of all probes specific for the target genes (Table S1). After the hybridizations step, samples were washed to remove excess probes and fluorescently tagged in a two-step color development process. Regions of interest were imaged as described below and fluorescent signals removed during decolorization. Color development, imaging and decolorization were repeated for multiple cycles to build a unique combinatorial code for every target gene that was derived from raw images as described below.

**Probe design**

The probes for 100 genes were designed using Resolve’s proprietary design algorithm. Briefly, the probe-design was performed at the gene-level. For every targeted gene all full-length protein coding transcript sequences from the ENSEMBL database were used as design targets if the isoform had the GENCODE annotation tag ‘basic’ ^1,2^.To speed up the process, the calculation of computationally expensive parts, especially the off-target searches, the selection of probe sequences was not performed randomly, but limited to sequences with high success rates. To filter highly repetitive regions, the abundance of k-mers was obtained from the background transcriptome using Jellyfish ^3^. Every target sequence was scanned once for all k-mers, and those regions with rare k-mers were preferred as seeds for full probe design. A probe candidate was generated by extending a seed sequence until a certain target stability was reached. A set of simple rules was applied to discard sequences that were found experimentally to cause problems. After these fast screens, every kept probe candidate was mapped to the background transcriptome using ThermonucleotideBLAST ^4^ and probes with stable off-target hits were discarded. Specific probes were then scored based on the number of on-target matches (isoforms), which were weighted by their associated APPRIS level ^5^, favoring principal isoforms over others. A bonus was added if the binding-site was inside the protein-coding region. From the pool of accepted probes, the final set was composed by greedily picking the highest scoring probes.

Samples were imaged on a Zeiss Celldiscoverer 7, using the 50x Plan Apochromat water immersion objective with an NA of 1.2 and the 0.5x magnification changer, resulting in a 25x final magnification. Standard CD7 LED excitation light source, filters, and dichroic mirrors were used together with customized emission filters optimized for detecting specific signals. Excitation time per image was 1000 ms for each channel (DAPI was 20 ms). A z-stack was taken at each region with a distance per z-slice according to the Nyquist-Shannon sampling theorem. The custom CD7 CMOS camera (Zeiss Axiocam Mono 712, 3.45 µm pixel size) was used. For each region, a z-stack per fluorescent color (two colors) was imaged per imaging round. A total of 8 imaging rounds were done for each position, resulting in 16 z-stacks per region. The completely automated imaging process per round (including water immersion generation and precise relocation of regions to image in all three dimensions) was realized by a custom python script using the scripting API of the Zeiss ZEN software (Open application development).

**Spot Segmentation**

The algorithms for spot segmentation were written in Java and are based on the ImageJ library functionalities. Only the iterative closest point algorithm is written in C++ based on the libpointmatcher library (<https://github.com/ethz-asl/libpointmatcher>).

**Preprocessing**

First, background fluorescence was corrected in all images. A target value for the allowed number of maxima was determined based upon the area of the slice in µm² multiplied by the factor 0.5 (empirically optimized). The brightest maxima per plane were determined, based upon an empirically optimized threshold. The number and location of the respective maxima was stored. This procedure was done for every image slice independently. Maxima that did not have a neighboring maximum in an adjacent slice (called z-group) were excluded. The resulting maxima list was further filtered in an iterative loop by adjusting the allowed thresholds for (Babs-Bback) and (Bperi-Bback) to reach a feature target value (Babs: absolute brightness, Bback: local background, Bperi: background of periphery within 1 pixel). This feature target values were based upon the volume of the 3D-image. Only maxima still in a zgroup of at least 2 after filtering were passing the filter step. Each z-group was counted as one hit. The members of the z-groups with the highest absolute brightness were used as features and written to a file. They resemble a 3D-point cloud. Final signal segmentation and decoding: To align the raw data images from different imaging rounds, images had to be corrected. To do so, the extracted feature point clouds were used to find the transformation matrices. For this purpose, an iterative closest point cloud algorithm was used to minimize the error between two point-clouds. The point clouds of each round were aligned to the point cloud of round one (reference point cloud). The corresponding point clouds were stored for downstream processes. Based upon the transformation matrices the corresponding images were processed by a rigid transformation using trilinear interpolation. The aligned images were used to create a profile for each pixel consisting of 16 values (16 images from two color channels in 8 imaging rounds). The pixel profiles were filtered for variance from zero normalized by total brightness of all pixels in the profile. Matched pixel profiles with the highest score were assigned as an ID to the pixel. Pixels with neighbors having the same ID were grouped. The pixel groups were filtered by group size, number of direct adjacent pixels in group, number of dimensions with size of two pixels. The local 3D-maxima of the groups were determined as potential final transcript locations. Maxima was filtered by number of maxima in the raw data images where a maximum was expected. Remaining maxima were further evaluated by the fit to the corresponding code. The remaining maxima were written to the results file and considered to resemble transcripts of the corresponding gene. The ratio of signals matching to codes used in the experiment and signals matching to codes not used in the experiment were used as estimation for specificity (false positives).

**Table S1.** Genes analyzed from human midbrain samples with Molecular Cartography technology ™ (Resolve Biosciences).

| **No** | **CatNo** | **Species** | **Gene** | **Target** |
| --- | --- | --- | --- | --- |
| 1 | P0M1F | Hs | MS4A7 | ENSG00000166927 |
| 2 | P0T18 | Hs | SELPLG | ENSG00000110876 |
| 3 | P0W16 | Hs | ACADM | ENSG00000117054 |
| 4 | P1V18 | Hs | GPX8 | ENSG00000164294 |
| 5 | P2V19 | Hs | STAT2 | ENSG00000170581 |
| 6 | P2W18 | Hs | SERPINH1 | ENSG00000149257 |
| 7 | P3V1A | Hs | CCL2 | ENSG00000108691 |
| 8 | P3W19 | Hs | ACADL | ENSG00000115361 |
| 9 | P4T1D | Hs | KDR | ENSG00000128052 |
| 10 | P4V1C | Hs | IFI44 | ENSG00000137965 |
| 11 | P4W1A | Hs | ACADS | ENSG00000122971 |
| 12 | P5T1E | Hs | IRF8 | ENSG00000140968 |
| 13 | P5V1D | Hs | ARG1 | ENSG00000118520 |
| 14 | P5W1C | Hs | ACADSB | ENSG00000196177 |
| 15 | P6T1F | Hs | SCARB1 | ENSG00000073060 |
| 16 | P6V1E | Hs | MRC1 | ENSG00000260314 |
| 17 | P6W1D | Hs | RUNX2 | ENSG00000124813 |
| 18 | P7T1G | Hs | MAF | ENSG00000178573 |
| 19 | P7V1F | Hs | TNF | ENSG00000232810 |
| 20 | P8V1G | Hs | NFKB1 | ENSG00000109320 |
| 21 | P9V1H | Hs | PELI1 | ENSG00000197329 |
| 22 | P9W1G | Hs | FOS | ENSG00000170345 |
| 23 | PAP9Z | Hs | MOG | ENSG00000204655 |
| 24 | PAV1J | Hs | CD33 | ENSG00000105383 |
| 25 | PC913 | Hs | BMP2 | ENSG00000125845 |
| 26 | PCN90 | Hs | PRDX6 | ENST00000340385 |
| 27 | PCP9L | Hs | CAT | ENST00000241052 |
| 28 | PCT1M | Hs | ADGRE5 | ENSG00000123146 |
| 29 | PCV1K | Hs | PILRB | ENSG00000121716 |
| 30 | PDP90 | Hs | SYT11 | ENST00000368324 |
| 31 | PDV1M | Hs | NUP160 | ENSG00000030066 |
| 32 | PEC57 | Hs | SNAP25 | ENSG00000132639 |
| 33 | PEP91 | Hs | ELAVL4 | ENSG00000162374 |
| 34 | PEV1N | Hs | LRRK2 | ENSG00000188906 |
| 35 | PEW1M | Hs | RELA | ENSG00000173039 |
| 36 | PFP92 | Hs | PPARGC1A | ENSG00000109819 |
| 37 | PFT1Q | Hs | FCGR3A | ENSG00000203747 |
| 38 | PFV1P | Hs | RGS1 | ENSG00000090104 |
| 39 | PFW1N | Hs | CEBPA | ENSG00000245848 |
| 40 | PGN94 | Hs | LYNX1 | ENSG00000180155 |
| 41 | PGP93 | Hs | SLITRK4 | ENSG00000179542 |
| 42 | PGT1R | Hs | CD80 | ENSG00000121594 |
| 43 | PHN95 | Hs | PSMD5 | ENSG00000095261 |
| 44 | PHT1S | Hs | CD86 | ENSG00000114013 |
| 45 | PHV1R | Hs | HLA-DRA | ENSG00000204287 |
| 46 | PHW1Q | Hs | EGR1 | ENSG00000120738 |
| 47 | PHW3S | Hs | STAT3 | ENSG00000168610 |
| 48 | PJN96 | Hs | SCRN1 | ENSG00000136193 |
| 49 | PJT1T | Hs | S1PR1 | ENSG00000170989 |
| 50 | PJV1S | Hs | BIN1 | ENSG00000136717 |
| 51 | PKN97 | Hs | MID1IP1 | ENSG00000165175 |
| 52 | PKT1V | Hs | MEF2C | ENSG00000081189 |
| 53 | PKV1T | Hs | PLCG2 | ENSG00000197943 |
| 54 | PKW1S | Hs | FOSB | ENSG00000125740 |
| 55 | PM04R | Hs | S100A6 | ENSG00000197956 |
| 56 | PMF16 | Hs | MAP2 | ENSG00000078018 |
| 57 | PMT1W | Hs | WASF2 | ENSG00000158195 |
| 58 | PMV1V | Hs | MS4A4A | ENSG00000110079 |
| 59 | PMW1T | Hs | JUN | ENSG00000177606 |
| 60 | PNT1X | Hs | AKT1 | ENSG00000142208 |
| 61 | PNV1W | Hs | SREBF1 | ENSG00000072310 |
| 62 | PPT1Y | Hs | APAF1 | ENSG00000120868 |
| 63 | PPV1X | Hs | MTIF3 | ENSG00000122033 |
| 64 | PPW1W | Hs | KLF4 | ENSG00000136826 |
| 65 | PQN9C | Hs | CTSF | ENST00000310325 |
| 66 | PQT1Z | Hs | NOX1 | ENSG00000007952 |
| 67 | PQV1Y | Hs | DNM1L | ENSG00000087470 |
| 68 | PQW1X | Hs | SALL1 | ENSG00000103449 |
| 69 | PRJ17 | Hs | CX3CR1 | ENSG00000168329 |
| 70 | PRN9D | Hs | OLR1 | ENSG00000173391 |
| 71 | PRT1L | Hs | CYBB | ENSG00000165168 |
| 72 | PRW1Y | Hs | MMP9 | ENSG00000100985 |
| 73 | PSK17 | Hs | ITGAL | ENSG00000005844 |
| 74 | PSV1L | Hs | RAB5A | ENSG00000144566 |
| 75 | PSW1Z | Hs | MEF2A | ENSG00000068305 |
| 76 | PT71M | Hs | BCL2 | ENSG00000171791 |
| 77 | PTT11 | Hs | NOX4 | ENSG00000086991 |
| 78 | PTV10 | Hs | RHOB | ENSG00000143878 |
| 79 | PVS13 | Hs | GAS6 | ENSG00000183087 |
| 80 | PVT12 | Hs | NOX5 | ENSG00000255346 |
| 81 | PVV11 | Hs | PIEZO1 | ENSG00000103335 |
| 82 | PVW10 | Hs | MTF1 | ENSG00000188786 |
| 83 | PVZ40 | Hs | AQP4 | ENSG00000171885 |
| 84 | PWS14 | Hs | MERTK | ENSG00000153208 |
| 85 | PWT13 | Hs | PRDX2 | ENSG00000167815 |
| 86 | PWZ41 | Hs | AQP9 | ENSG00000103569 |
| 87 | PX71Q | Hs | CFLAR | ENSG00000003402 |
| 88 | PXS15 | Hs | PROS1 | ENSG00000184500 |
| 89 | PXT14 | Hs | SOD2 | ENSG00000112096 |
| 90 | PXV13 | Hs | ENTPD1 | ENSG00000138185 |
| 91 | PXW12 | Hs | KLF2 | ENSG00000127528 |
| 92 | PXZ42 | Hs | TH | ENSG00000180176 |
| 93 | PYC5R | Hs | CNP | ENSG00000173786 |
| 94 | PYS16 | Hs | P2RY12 | ENSG00000169313 |
| 95 | PYT15 | Hs | SOD3 | ENSG00000109610 |
| 96 | PYZ43 | Hs | GFAP | ENSG00000131095 |
| 97 | PZS17 | Hs | TMEM119 | ENSG00000183160 |
| 98 | PZT16 | Hs | GPX3 | ENSG00000211445 |
| 99 | PZV15 | Hs | ABCA6 | ENSG00000154262 |
| 100 | PZZ44 | Hs | BMP4 | ENSG00000125378 |

**References**

1. Frankish, A. *et al.* GENCODE reference annotation for the human and mouse genomes. *Nucleic Acids Res* **47**, (2019).

2. Yates, A. D. *et al.* Ensembl 2020. *Nucleic Acids Res* **48**, (2020).

3. Marçais, G. & Kingsford, C. A fast, lock-free approach for efficient parallel counting of occurrences of k-mers. *Bioinformatics* **27**, (2011).

4. Gans, J. D. & Wolinsky, M. Improved assay-dependent searching of nucleic acid sequence databases. *Nucleic Acids Res* **36**, (2008).

5. Rodriguez, J. M. *et al.* APPRIS 2017: Principal isoforms for multiple gene sets. *Nucleic Acids Res* **46**, (2018).

**Supplementary data**

RNA sequencing data and spatial transcriptomics generated in this study is available in Zenodo through the link:

<https://zenodo.org/record/7803015?token=eyJhbGciOiJIUzUxMiIsImV4cCI6MTcxMjI2Nzk5OSwiaWF0IjoxNjgwNzY0Mzk4fQ.eyJkYXRhIjp7InJlY2lkIjo3ODAzMDE1fSwiaWQiOjMxNzE4LCJybmQiOiJhMDg5OGIxZCJ9.umY2ZA8l18DFCokp0k9LdBKLQEsxE5FPs3i_wYW2tXCPR8LUAgZaWlypvyFukijSMwAJkEhL_Xt9iPNReOrhyw>

**Supplementary full-sized Western blots**

**
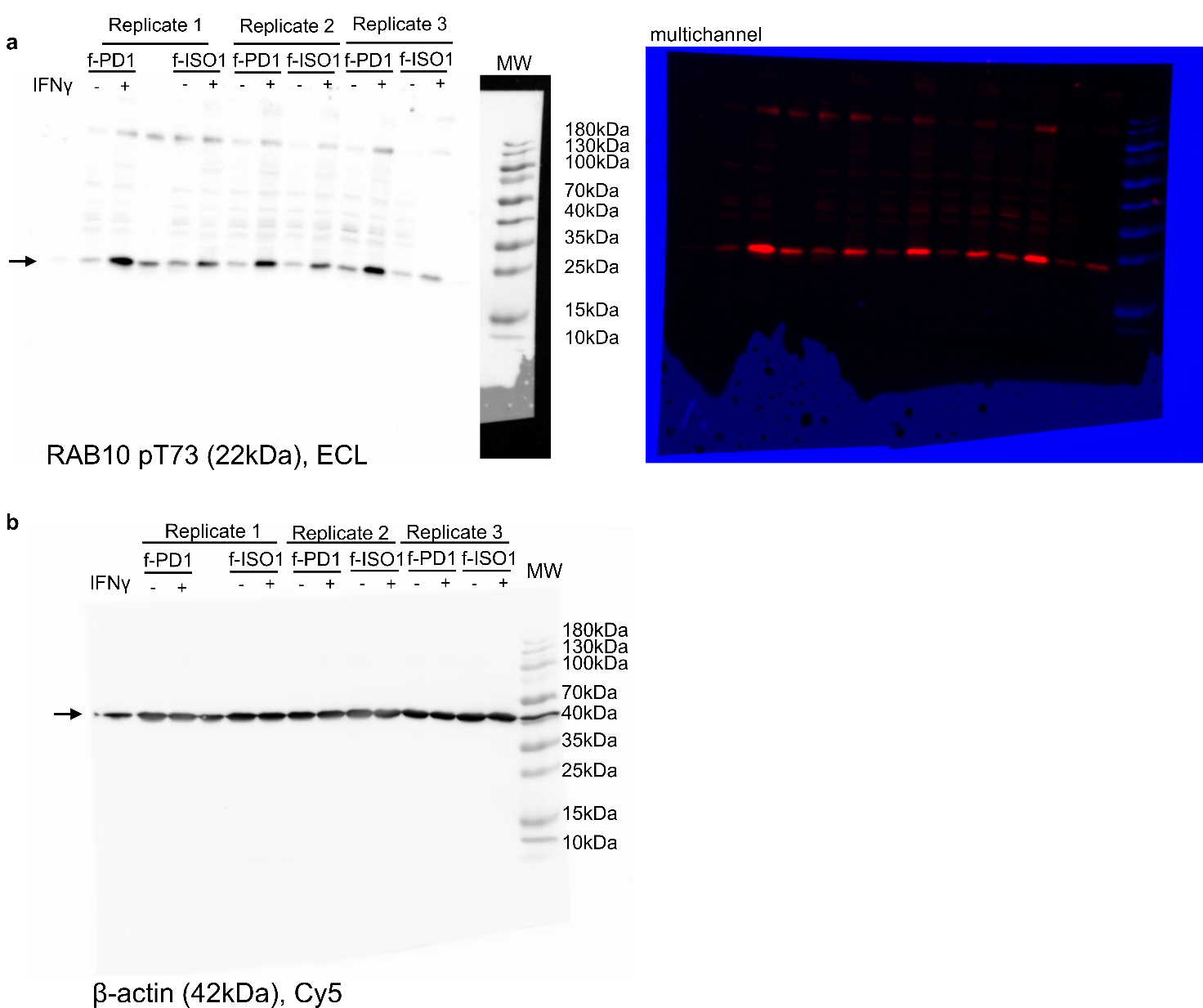
**

**Supplementary full-sized Western blot (RAB10 pT73)**. Original full sized Western blots a) phosphorylation of LRRK2 target RAB10 (pT73) compared to b) β-actin. Multichannel image shows RAB10 pT73 (red) and molecular weight (MW, blue) marker on the same blot. Proteins were extracted from vehicle or IFNγ stimulated iMGL and run in parallel from 3 batches of iMGL.

**
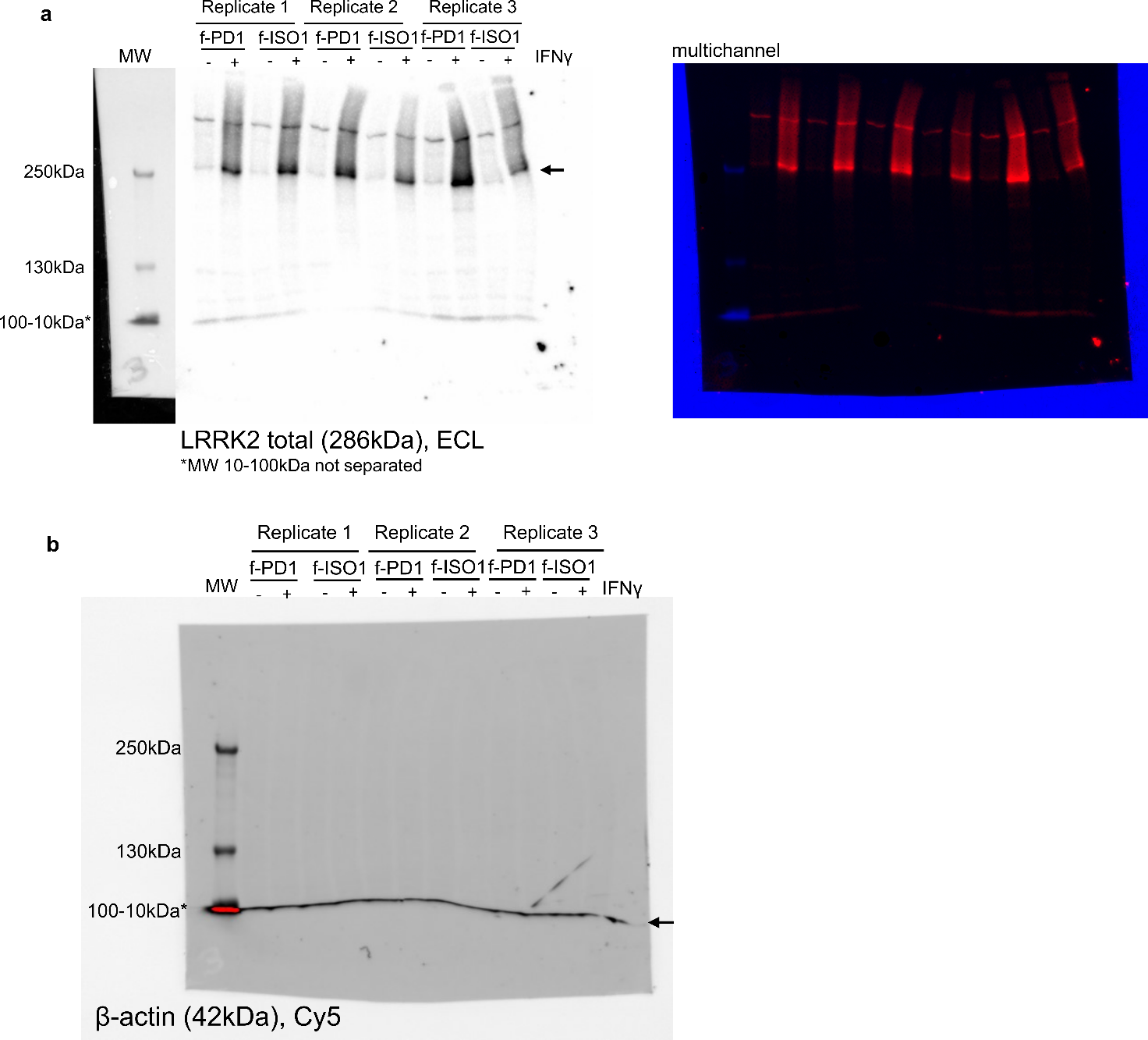
**

**Supplementary full-sized Western blot (total LRRK2)** a) Expression of LRRK2 compared to b) β-actin. Multichannel image shows LRRK2 (red) and MW-marker (blue) on the same blot. Proteins were extracted from vehicle or IFNγ stimulated iMGL and run in parallel from 3 batches of iMGL.

**Supplementary figure legends**

**Supplementary figure 1.** **Characterization of iMGL with RNAseq** a) f-ISO1 and f-PD1 iPSC show normal karyotype b) Correlation of transcriptional profiles between our iMGL, iPSC and (iPSC-)microglia and monocytes from Abud et al dataset (2017) ^29^. c-d) Western blot analysis of expression of LRRK2 and phosphorylation of LRRK2 target RAB10 from vehicle and IFNγ stimulated iMGL. c) Shared pathways defining PD-microglia, analysed from DEGs between f‑PD1/f-ISO1 IFNγ stimulated iMGL and PD/Control microglia from the snSEQ of midbrain dataset from Smajić et al (2022) For iMGL, n=3-4 batches of iMGL, with 1-2 million cells used for each batch, n=2 independently collected batches for both iPSC genotypes. For snSEQ, more details can be found in the original publications 30, n=6 controls (22 433 nuclei in total), n=5 idiopathic PD patients (19 002 nuclei in total).

**Supplementary Figure 2. Characterization of iMGL.** a) Expression of microglia specific marker genes analyzed with qPCR for m-CTRL and m-PD, n=4 independent batches of iMGL. b) Representative images from microglia marker staining from (f-ISO1) iMGL, scale bar 20 µm. Well without primary antibody was used as staining control (Secondary antibody only, Sec.Ab) c) Representative curves of Ca2+ response to ATP or ADP application measured with Fluo-4. d) Percentage of Ca2+ responsive cells and e) the ratio of nucleoside to ionomycin evoked amplitude, data measured from 4 independent batches of iMGL (n=4) for m-CTLR and m-PD, and 6 independent batches of iMGL (n=6) for f-ISO1, f-PD1. The number of cells analysed for ATP: 870,1472, 2719, 2456 respectively, and for ADP: 681, 901, 1011, 3008 respectively. Data are represented as mean±SD, Two-way Anova with Sidak multiple comparison, p-value *<0.5, **<0.01.

**Supplementary Figure 3. Mitochondrial functionality and membrane potential measured from iMGL.** Energy phenotype of a) isogenic iMGL and b) male iMGL presented as OCR/ECAR kinetic graph from "basal" state to "stressed" state calculated from Mito Stress test. c-d) Mitochondrial parameters from Mito Stress assay for isogenic and male iMGL in Vehicle and IFNγ conditions. Data presented as mean±SD, n=3 for isogenic pair, and n=4 for male pair. e) Representative trace of Rho123 fluorescence after addition of 4 µM FCCP (60 sec.). f) Basal fluorescence intensity and FCCP evoked response in Rho123 fluorescence intensity in each genotype. Data represented as mean±SD, n=6 independent batches for f-ISO1/f-PD1 and n=3 independent batches for m-CTRL/m-PD. g) Representative Rho123 traces upon IFNγ stimulation. Effect of IFNγ in isogenic iMGL to h) basal fluorescence intensity and on FCCP-evoked mitochondrial depolarization. Data represented as mean±SD, n=3 independent batches. Numbers of cells analyzed m-CTRL 1706, m-PD 3780. f-ISO1 (Veh/IFNγ) 3142/971, f-PD1 (Veh/IFNγ) 2850/943. Data is analyzed with Two-way ANOVA with Sidak multiple comparison, p-value ***≤ 0.001.

**Supplementary Figure 4. Microglial spatial transcriptional profile in human midbrain post-mortem samples**. The transcript expression of 100-plex panel was detected with Molecular Cartography method from human midbrain samples. a) GFAP contributes 49-89% of the detected transcripts and is widely spread in the tissue. Scale bar 200 µm. b) Annotated cell populations in the control and PD midbrain samples c) Expression patterns of genes in the annotated cell populations. d) Average expression of genes which were differentially expressed between control and non-consensus PD-microglia. For the midbrain samples, n=8 controls, n=7 PD patients, one section per sample. Data presented as distribution percentage of average expression between the microglial classes, differential expression analyzed with the Wilcoxon rank sum test.

**Supplementary Table S1. RNAseq analysis of iMGL: quality control and differentially expressed genes.** Impact of LRRK2-G2019S mutation in iMGL was analyzed with RNAseq**.**

**Supplementary Table S2. Functional annotations of DEGs on IPA (bulk RNAseq of iMGL).** Impact of LRRK2-G2019S mutation in iMGL was analyzed with RNAseq. Predicted functional annotation of differentially expressed genes was anlyzed with Ingenuity pathway analysis (IPA).

**Supplementary Table S3. PD-microglia signature pathways annotated by IPA.** Shared pathways describing PD-microglia signature from bulk RNAseq of iMGL carrying LRRK2-G2019S and snSEQ from human midbrain microglia.

**Supplementary Table S4. Average expression and distribution of genes in microglia from human midbrain samples detected by Molecular Cartography.** Human microglial expression of multiplex panel was analyzed from post-mortem midbrain samples from idiopathic PD cases and healthy controls.
