# Supplementary figures and images for: Human iPSC-derived microglia carrying the LRRK2-G2019S mutation show a Parkinson’s disease related transcriptional profile and function
Source: Sci Rep. 2023 Dec 13;13:22118. doi: 10.1038/s41598-023-49294-9 (PMC10719377; doi:10.1038/s41598-023-49294-9)

a

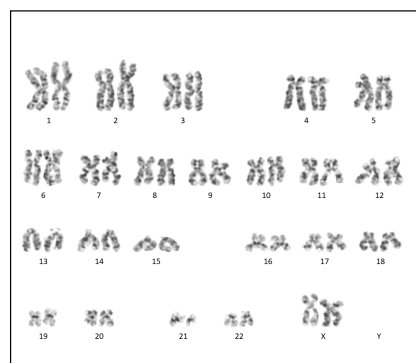

f-ISO1

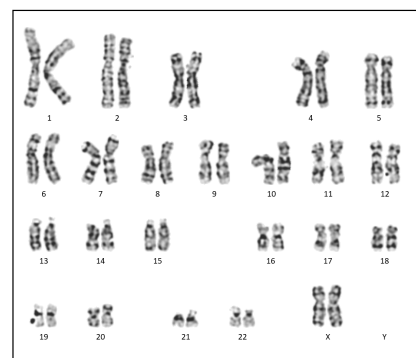

f-PD1

b

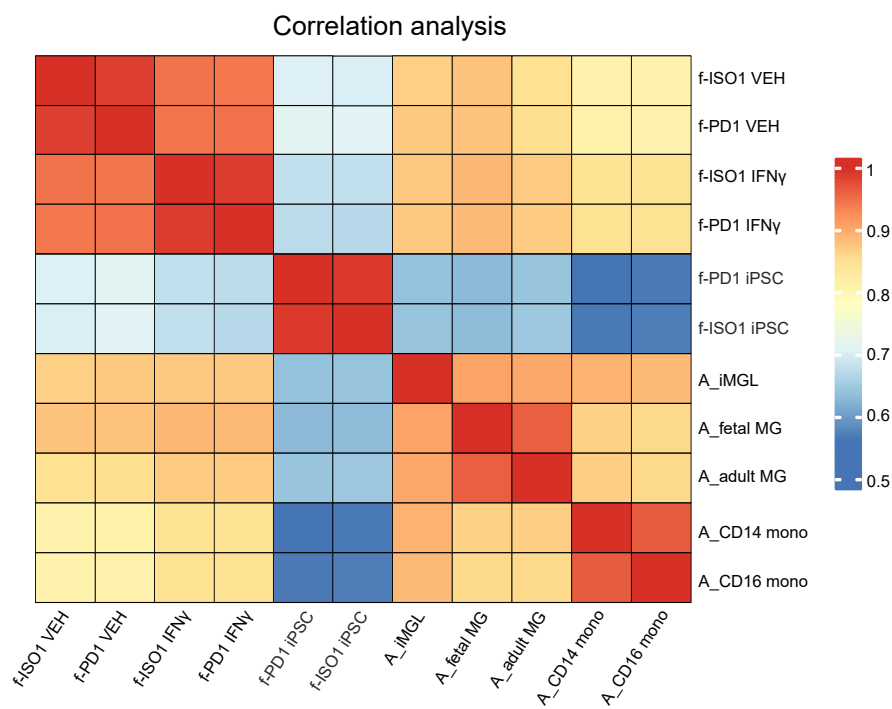

c

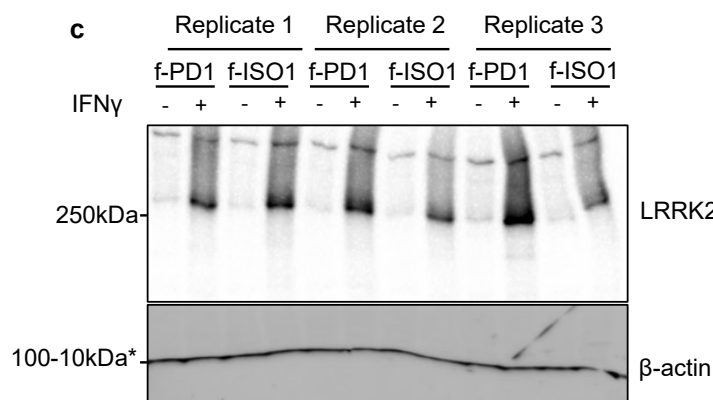

d

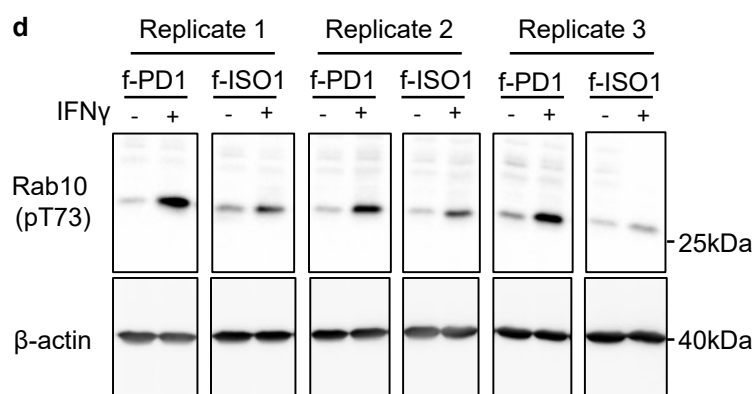

e

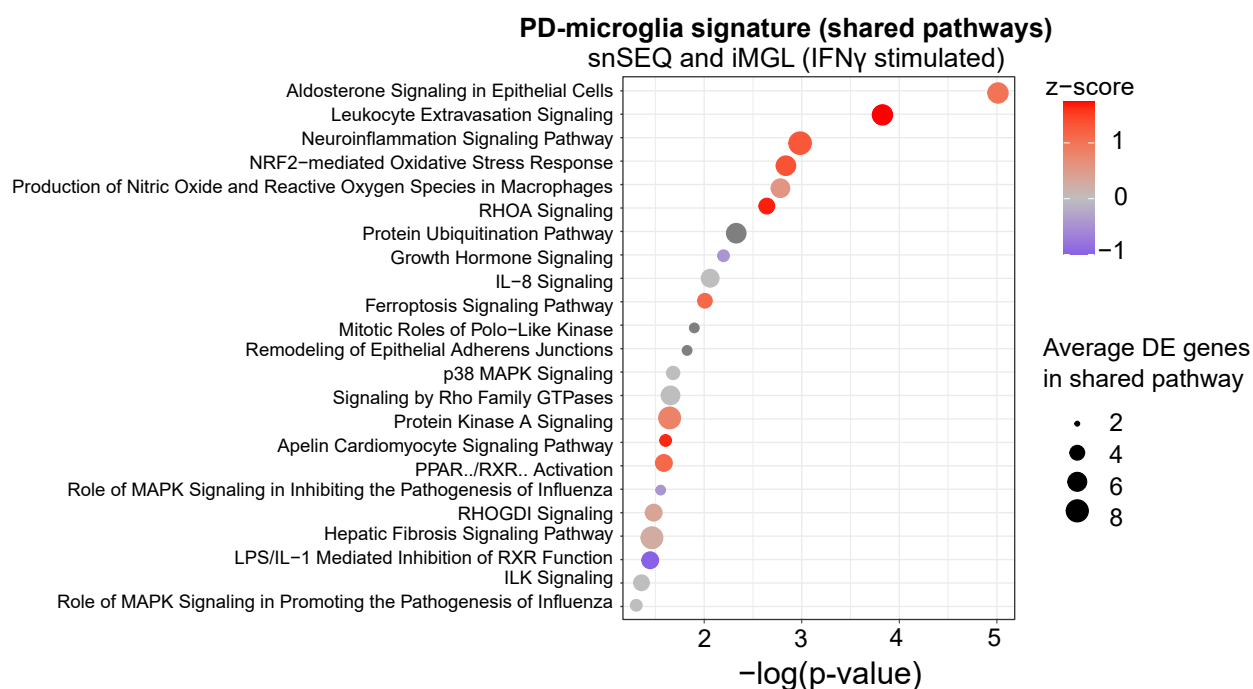

Supplement: Supplementary file 2 — Supplementary Figure S1. [file 41598_2023_49294_MOESM2_ESM.pdf]

**a**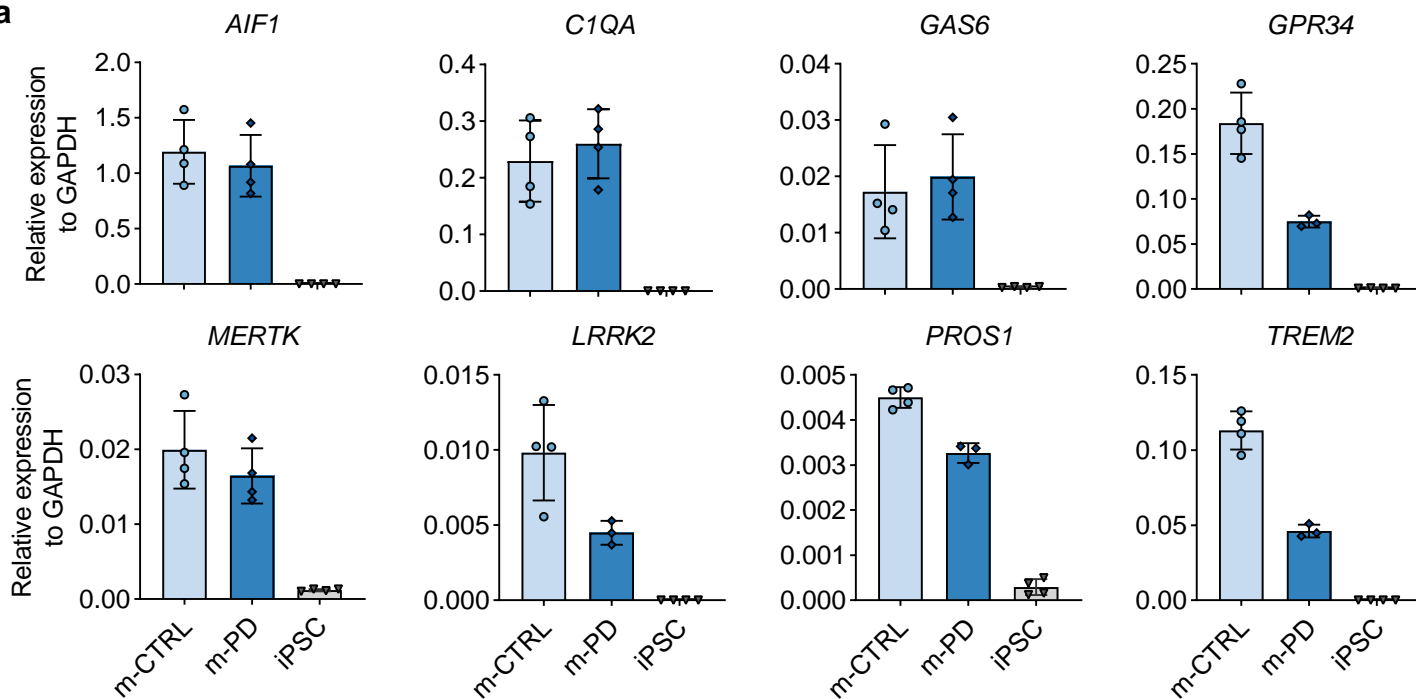**b**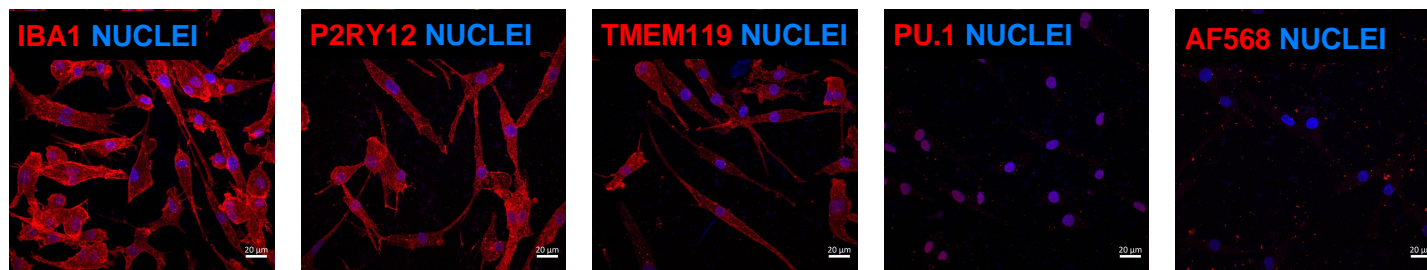**c**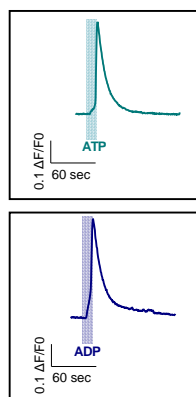**d**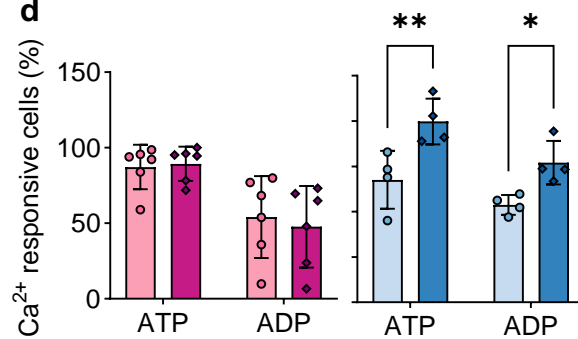**e**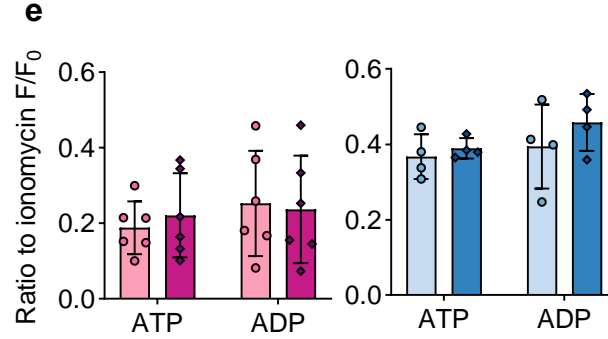

f-ISO1 f-PD1 m-CTRL m-PD

Supplement: Supplementary file 3 — Supplementary Figure S2. [file 41598_2023_49294_MOESM3_ESM.pdf]

# imGL Energy Phenotype

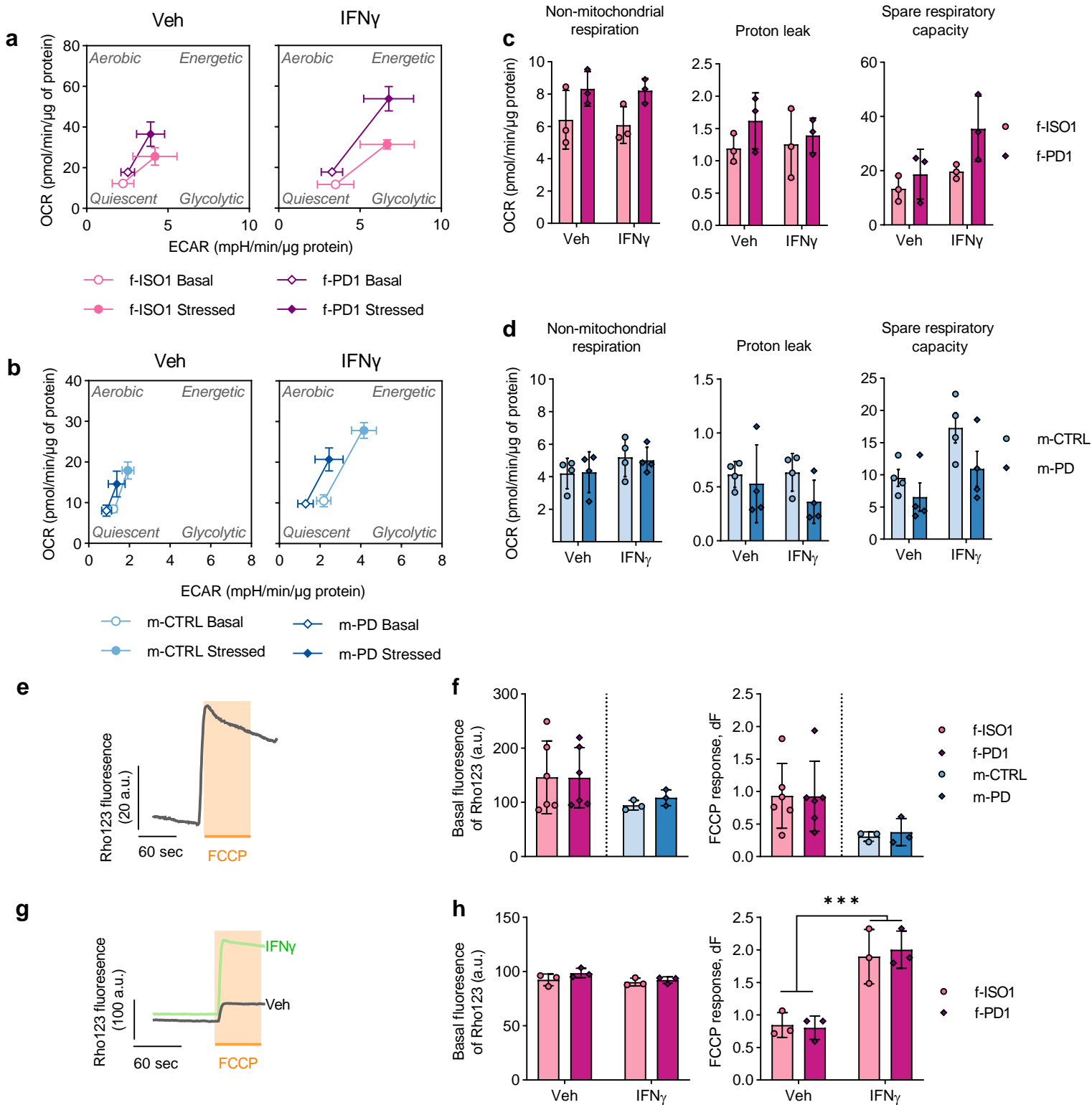

Supplement: Supplementary file 4 — Supplementary Figure S3. [file 41598_2023_49294_MOESM4_ESM.pdf]

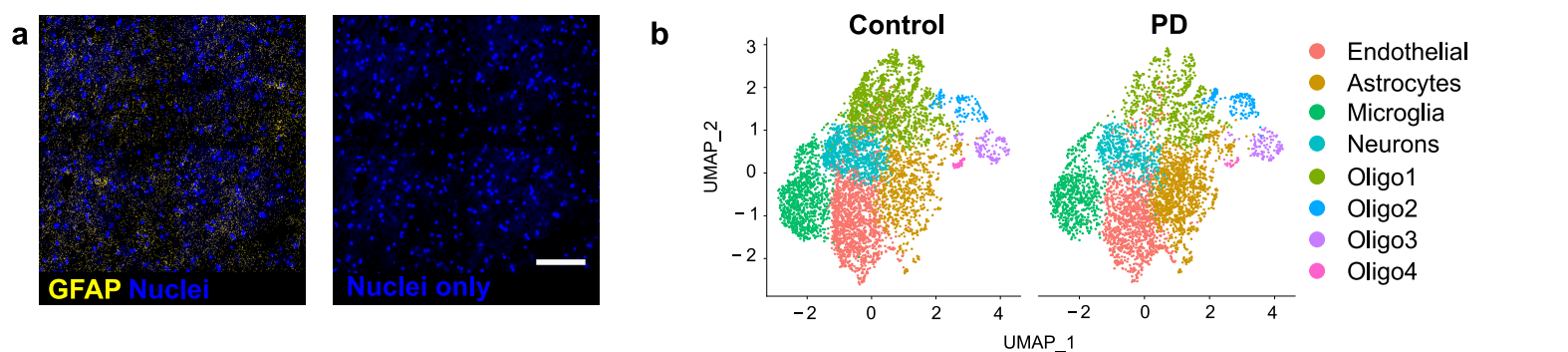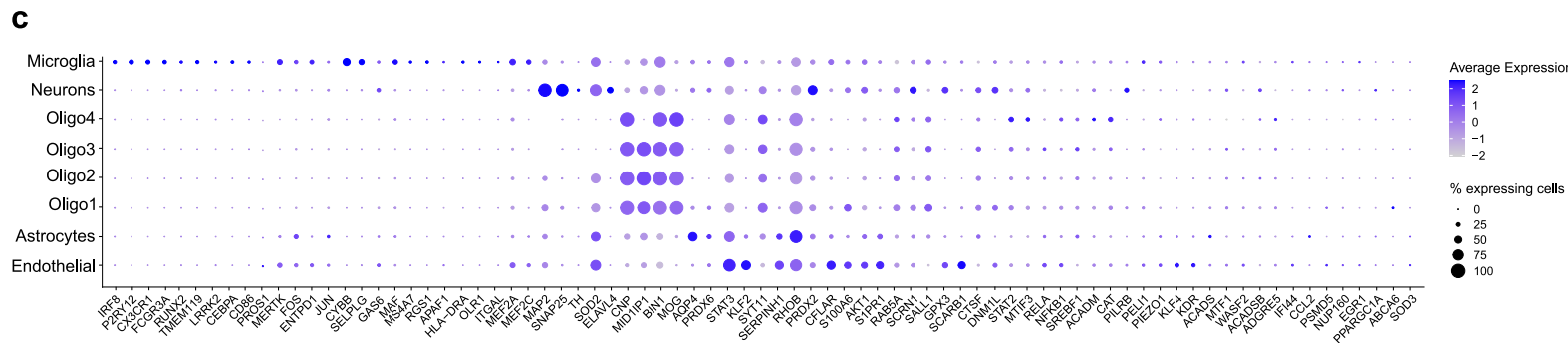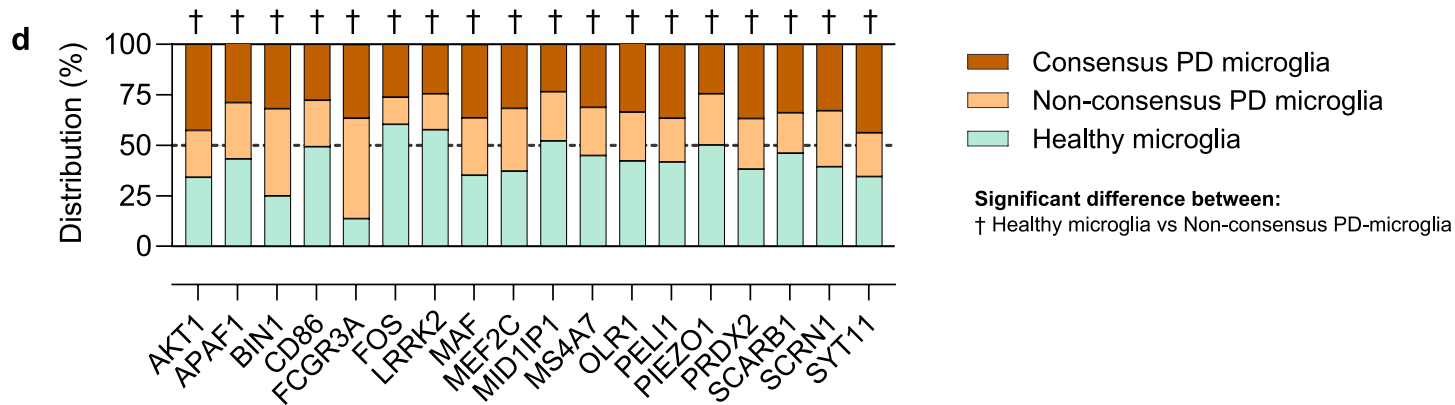

Supplement: Supplementary file 5 — Supplementary Figure S4. [file 41598_2023_49294_MOESM5_ESM.pdf]
